# Cell‐free chromatin immunoprecipitation can determine tumor gene expression in lung cancer patients

**DOI:** 10.1002/1878-0261.13394

**Published:** 2023-03-05

**Authors:** Christoffer Trier Maansson, Peter Meldgaard, Magnus Stougaard, Anders Lade Nielsen, Boe Sandahl Sorensen

**Affiliations:** ^1^ Department of Clinical Biochemistry, Faculty of Health Aarhus University Hospital Denmark; ^2^ Department of Clinical Medicine Aarhus University Denmark; ^3^ Department of Biomedicine Aarhus University Denmark; ^4^ Department of Oncology Aarhus University Hospital Denmark; ^5^ Department of Pathology Aarhus University Hospital Denmark

**Keywords:** cell‐free ChIP, epigenetics, gene expression, liquid biopsy, non‐small‐cell lung cancer, small‐cell lung cancer

## Abstract

Cell‐free DNA (cfDNA) in blood plasma can be bound to nucleosomes that contain post‐translational modifications representing the epigenetic profile of the cell of origin. This includes histone H3 lysine 36 trimethylation (H3K36me3), a marker of active transcription. We hypothesised that cell‐free chromatin immunoprecipitation (cfChIP) of H3K36me3‐modified nucleosomes present in blood plasma can delineate tumour gene expression levels. H3K36me3 cfChIP followed by targeted NGS (cfChIP‐seq) was performed on blood plasma samples from non‐small‐cell lung cancer (NSCLC) patients (NSCLC, *n* = 8), small‐cell lung cancer (SCLC) patients (SCLC, *n* = 4) and healthy controls (*n* = 4). H3K36me3 cfChIP‐seq demonstrated increased enrichment of mutated alleles compared with normal alleles in plasma from patients with known somatic cancer mutations. Additionally, genes identified to be differentially expressed in SCLC and NSCLC tumours had concordant H3K36me3 cfChIP enrichment profiles in NSCLC (sensitivity = 0.80) and SCLC blood plasma (sensitivity = 0.86). Findings here expand the utility of cfDNA in liquid biopsies to characterise treatment resistance, cancer subtyping and disease progression.

## Graphical abstract

Blood plasma contains H3K36me3‐modified nucleosomes representing active genes in the cell of origin. Our findings show that cell‐free chromatin immunoprecipitation (cfChIP) of H3K36me3‐nucleosomes in plasma from lung cancer patients can reveal tumour gene expression. cfChIP followed by next‐generation sequencing identifies genes that are differentially expressed in the tumour of non‐small‐cell and small‐cell lung cancer patients. Created with BioRender.com.

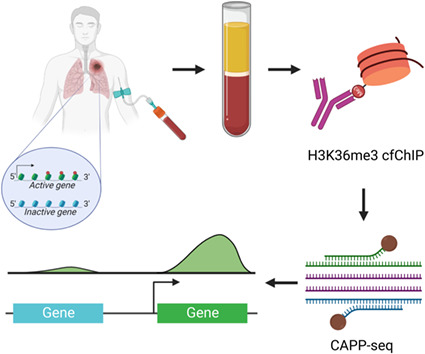

**Abbreviations**
AUC: area under the curve
CAPP‐seq: cancer personalised profiling by deep sequencing
cfChIP: cell‐free chromatin immunoprecipitation
cfChIP‐seq: cell‐free chromatin immunoprecipitation sequencing
cfDNA: cell‐free DNA
cfRNA: cell‐free RNA
ChIP: chromatin immunoprecipitation
ctDNA: circulating tumour DNA
ddPCR: droplet digital PCR
EGFR: epidermal growth factor receptor
EMT: epithelial‐mesenchymal transition
ENA: European Nucleotide Archive
FDR: false discovery rate
GEO: gene expression omnibus
H3K36me3: histone H3 lysine 36 trimethylation
H3K4me3: histone H3 lysine 4 trimethylation
log_2_(FC): log_2_ fold‐change
MAF: mutational allele fraction
miRNA: microRNA
NGS: next‐generation sequencing
NSCLC: non‐small‐cell lung cancer
PBMC: peripheral blood mononuclear cell
RNA‐seq: RNA sequencing
ROC: receiver operating characteristics
SCLC: small‐cell lung cancer
STR: Short Tandem Repeats
TPM: transcripts per million
TSS: transcription start site
UMAP: uniform manifold approximation and projection

## 1 Introduction

In recent years, studies have demonstrated how liquid biopsies can be used to study tumour burden and disease progression through quantification of tumour mutations [1, 2, 3, 4]. Typically, the circulating tumour DNA (ctDNA) in the total pool of cell‐free DNA (cfDNA) is analysed for the presence of mutations using, for example droplet digital PCR (ddPCR) [5, 6] or next‐generation sequencing (NGS) [7, 8]. However, the existence of a mutation in a gene do not necessarily reflect the gene expression status of the gene, and nonmutated genes can also have altered expression that supports tumour growth. Moreover, ctDNA has also been used to study the presence of treatment resistance mutations [1, 9] and identifying novel resistance mechanisms [10, 11, 12]. However, cancer therapy resistance is not always caused by the development of new mutations but could be caused by changes in gene expression mediated by, for example, epigenetic dysregulation [13]. While tumour gene expression can be analysed in tissue samples using RNA‐sequencing (RNA‐seq) [14] or even single‐cell RNA‐seq [15, 16], it is not always possible to acquire the necessary tumour tissue. In addition, an inherent problem with tissue biopsies is the heterogeneity within individual tumours and between different tumours in the same patient [17]. Therefore, increased efforts have been made into studying tumour gene expression based on non‐invasive liquid biopsies [18, 19, 20, 21, 22, 23, 24, 25].

In the cell nuclei, the genomic DNA is wrapped around histone proteins to generate the nucleosome core particle [26]. The interaction between DNA and histones is maintained following the release of cfDNA to the blood [27], and thereby cfDNA can be circulating as nucleosomes [28, 29, 30]. The epigenetic profile, such as post‐translational modifications of histones, from the cell of origin can be kept in the blood [31, 32]. This includes histone modifications such as H3K4me3 and H3K36me3 that are associated with active transcription of a gene [33, 34]. This has led to the development of chromatin immunoprecipitation (ChIP)‐based protocols targeting these histone modifications in liquid biopsies in order to have a surrogate measure of tumour gene expression [18, 19, 20]. In relation to lung cancer, we have previously shown how cell‐free chromatin immunoprecipitation (cfChIP) followed by ddPCR can differentiate squamous cell carcinoma from adenocarcinomas based on *KRT6ABC* enrichment [19]. Furthermore, we have demonstrated that cfChIP followed by ddPCR targeting *epidermal growth factor receptor* (*EGFR*) variants can detect lung tumour‐specific expression of *EGFR* [20].

In this study, we examine whether cfChIP coupled with targeted NGS in a cfChIP‐seq procedure can be used to determine gene expression in tumours from lung cancer patients. We demonstrate that H3K36me3 ChIP‐seq correlate with RNA‐levels in lung cancer cell lines. Following this, we evaluate H3K36me3 gene enrichment profiles in both non‐small‐cell lung cancer (NSCLC) and small‐cell lung cancer (SCLC) patients. We identify differently cfChIP enriched genes including *EGFR*, *CRMP1* and *SMAD4* with known differential gene expression between the two tumour types that we verify using RNA‐seq data from NSCLC and SCLC cell lines.

## 2 Materials and methods

### 2.1 Lung cancer patients and blood samples

This study was performed in accordance with the Declaration of Helsinki and accepted by the Central Denmark Region Committee on Biomedical Research Ethics (No. 1‐10‐72‐83‐14). Written informed consent was obtained from all individuals. Twelve lung cancer patients with stage IV NSCLC (*n* = 8) or SCLC (*n* = 4) were included in this study. The NSCLC patients were either diagnosed with adenocarcinoma (*n* = 4) or squamous cell carcinoma (*n* = 4). Patient characteristics are described in Table S1. Peripheral blood was drawn from each patient and four healthy controls in 10 mL EDTA tubes and centrifuged within 2 h at 1400 ***g*** for 15 min at room temperature. Plasma was aliquoted and stored at −80 °C. All blood samples were taken before treatment of the lung cancer patient was initiated.

### 2.2 Cell cultures

NSCLC cell lines, A549 (RRID: CVCL_0023, ATCC, LCG Standards, Wesel, Germany) and HCC827 (RRID: CVCL_2063, ATCC, LCG Standards), were grown in RPMI medium with 10% foetal calf serum and 1% penicillin–streptomycin (Gibco, Thermo Fischer Scientific, Waltham, MA, USA). An erlotinib resistant clone of HCC827 (called HCC827‐MET) was developed through stepwise escalation of erlotinib, as described previously [35]. This cell line was grown similar to HCC827 and A549, but with the addition of 5 μm erlotinib, in order to avoid re‐growth of nonresistant HCC827 cells. All cells were cultivated in 5% CO_2_ at 37 °C and authenticated using the GenePrint 10 System (Promega, #B9510, Madison, WI, USA) according to the manufacturer's instructions within 3 years. In brief, Short Tandem Repeats (STR) profiles of 10 different loci were compared with available STR profiles from cell bank databases. All cell lines tested negative for mycoplasma contamination.

### 2.3 Data from public repositories

Data were acquired from lung cancer cell lines in the Cancer Cell Line Encyclopedia database [36]. A total of 123 NSCLC and 77 SCLC cell lines were identified. From these, 106 NSCLC and 50 SCLC cell lines had gene expression data available in 22Q2 release (Table S2). Furthermore, two gene expression data sets were acquired from the Gene Expression Omnibus (GEO): GSE179879 representing gene expression in NSCLC tumours (*n* = 18) [14] and GSE107011 representing gene expression in peripheral blood mononuclear cells (PBMCs) (*n* = 13) [37].

### 2.4 RNA purification and sequencing

Cellular RNA was purified from the same cell culture flask that was used for ChIP‐seq using the QIAamp RNA Blood Mini Kit (QIAGEN, Hilden, Germany). The quality and quantity of each RNA‐sample was estimated using an Agilent 2100 fragment analyzer (Agilent Technologies, Santa Clara, CA, USA) as described in Table S3. RNA‐seq was performed in biological triplicates using paired‐end 100 sequencing with DNBSeq at BGI Genomics, Hong‐Kong. Reads were filtered using SOAPnuke [38] by BGI Genomics, where adapters were trimmed and low quality as well as N reads were removed. Clean reads were aligned to hg38.p13 through hisat2 (v. 2.2.1) [39] and quantified with stringtie (v. 2.1.7) [40, 41] using the Galaxy platform [42].

### 2.5 Differential expression analysis

The normalised gene expression in transcripts per million (TPM) is presented as log_2_(TPM + 1) for each gene. Inactive, or very low expressed, genes with log_2_(TPM + 1) < 0.2 in both groups were excluded from the differential expression analysis. Differentially expressed genes between groups were identified with an absolute log_2_ fold‐change above 1 (log_2_(FC) > 1) and a *q*‐value < 0.05 based on a *t*‐test. The log_2_(FC) for each gene, *i*, between group *a* and group *b* is calculated as:

(1) $$\log_{2}{\left(\mathrm{FC}\right)}_{i}=\log_{2}\left(\bar{\mathrm{TP}M_{i}^{a}+1}\right)-\log_{2}\left(\bar{\mathrm{TP}M_{i}^{b}+1}\right).$$

$\bar{\mathrm{TP}M_{i}^{a}+1}$ and $\bar{\mathrm{TP}M_{i}^{b}+1}$ is the average TPM + 1 in gene, *i*, for group *a* and *b*, respectively.

### 2.6 Cell‐free and conventional chromatin immunoprecipitation

Both conventional ChIP and cfChIP was performed targeting H3K36me3 with anti‐H3K36me3 (Abcam 9050). Conventional ChIP was performed on biological triplicates of approximate 1.5 × 10^6^ cells as described in [19, 20] except that the NucleoSpin Gel and PCR Clean‐up kit (Macherey‐Nagel, Dueren, Germany) was used to purify both input and ChIP samples. CfChIP was performed with subtle modifications from previous publications [19, 20] on plasma samples from both healthy donors and cancer patients. Briefly, the undiluted plasma sample was cleared of circulating antibodies using 12.5 μL empty protein A/G magnetic beads (ThermoFisher Scientific, 88802) per mL plasma for 2 h at 4 °C. Subsequently 10 μL protein A/G magnetic beads were bound to 1 μg anti‐H3K36me3 antibodies (Abcam 9050) per mL plasma and added to the antibody cleared plasma to incubate over‐night at 4 °C. The antibody‐bead complexes were washed twice in ChIP wash buffer I (Tris–HCl 20 mm, NaCl 150 mm, EDTA 2 mm, Triton X‐100 1%, SDS 0.1%, pH = 8.0), twice in ChIP wash buffer II (Tris–HCl 20 mm, NaCl 350 mm, EDTA 2 mm, Triton X‐100 1%, SDS 0.1%, pH = 8.0) and once in TE buffer, followed by elution in elution buffer (Tris–HCl 10 mm, EDTA 1 mm, SDS 1%, pH = 8.0) for 1 h at 60 °C. Both the input and the cfChIP samples were purified using Apostle MiniMax High Efficiency cfDNA Isolation Kit (Beckman Coulter, Indianapolis, IN, USA) according to the manufacturer's instructions. The input plasma sample was used to estimate the cfDNA concentration using the Qubit dsDNA HS assay kit (Thermo Fisher Scientific) and the fragment lengths were analysed using an Agilent 2100 Bioanalyzer (Agilent Technologies). The amount of plasma used for cfChIP and input as well as the cfDNA concentration is described in Table S4.

### 2.7 Cancer personalised profiling by deep sequencing (CAPP‐seq)

ChIP (Table S5) and cfChIP samples were applied to CAPP‐seq [43] in order to discover gene expression patterns. Sequencing libraries were prepared with the AVENIO ctDNA surveillance kit (Roche Sequencing Solutions, Mannheim, Germany) using sample specific adaptors. One hundred and ninety‐seven lung cancer‐relevant genes were enriched using the AVENIO surveillance panel (Roche Sequencing Solutions) (Table S6) [44], which primarily captures coding regions of genes. The targeted gene fragments were sequenced using the NextSeq 500 (Illumina, San Diego, CA, USA) and the data were analysed with a modified version of the AVENIO Oncology Analysis Software to accommodate the low DNA input in immunoprecipitated samples. The software thresholds were lowered for minimal number reads (*n* = 1 000 000), average read depth (×10) as well as fraction of reads aligning to the genome (50%).

### 2.8 Gene enrichment quantification

Gene enrichment was estimated from the deduped BAM file of ChIP and cfChIP samples. ChIP gene enrichment was calculated using the following formula:

(2) $$\text{Enrichmen}t_{i}=\frac{N_{i}*10^{9}}{\sum_{i=1}^{197}N_{i}*k_{i}}$$

Where $N_{i}$ is the number of reads in gene *i* and $k_{i}$ is the coverage of gene *i*. $k_{i}$ is constant for each gene and is estimated by applying purified HCC827 DNA to CAPP‐seq and counting the number of bases in each target gene with a depth > 100 (Table S6).

cfChIP enrichment was normalised to the average cfChIP‐seq enrichment in four healthy individuals using the following formula:

(3) $${\text{Enrichment}}_{i}=\frac{\left(N_{i}-\overset{=}{H_{i}}\right)*10^{9}}{\left(\sum_{i=1}^{197}N_{i}-\bar{\sum_{i=1}^{197}H_{i}}\right)*k_{i}}$$

$\bar{H_{i}}$ is the average read count in gene *i* for healthy individuals and $\bar{\sum_{i=1}^{197}H_{i}}$ is the average number of total reads in healthy individuals.

Genes with increased enrichment between cell lines or patients with different tumour subtypes was identified using the following formula:

(4) $$\log_{2}{\left(\mathrm{FC}\right)}_{i}=\log_{2}\left(\bar{\text{Enrichmen}t_{i}^{a}}\right)-\log_{2}\left(\bar{\text{Enrichmen}t_{i}^{b}}\right)$$

Where $\bar{\text{Enrichmen}t_{i}^{a}}$ is the average enrichment of gene *i* for group *a* and $\bar{\text{Enrichmen}t_{i}^{b}}$ is the average enrichment of gene *i* for group *b*. Here a log_2_(FC) > 0 indicates increased enrichment in group *a* and log_2_(FC) < 0 indicates increased enrichment in group *b*.

## 3 Results

### 3.1 Correlation between mRNA expression and H3K36me3 ChIP enrichment

**Fig. 1:**
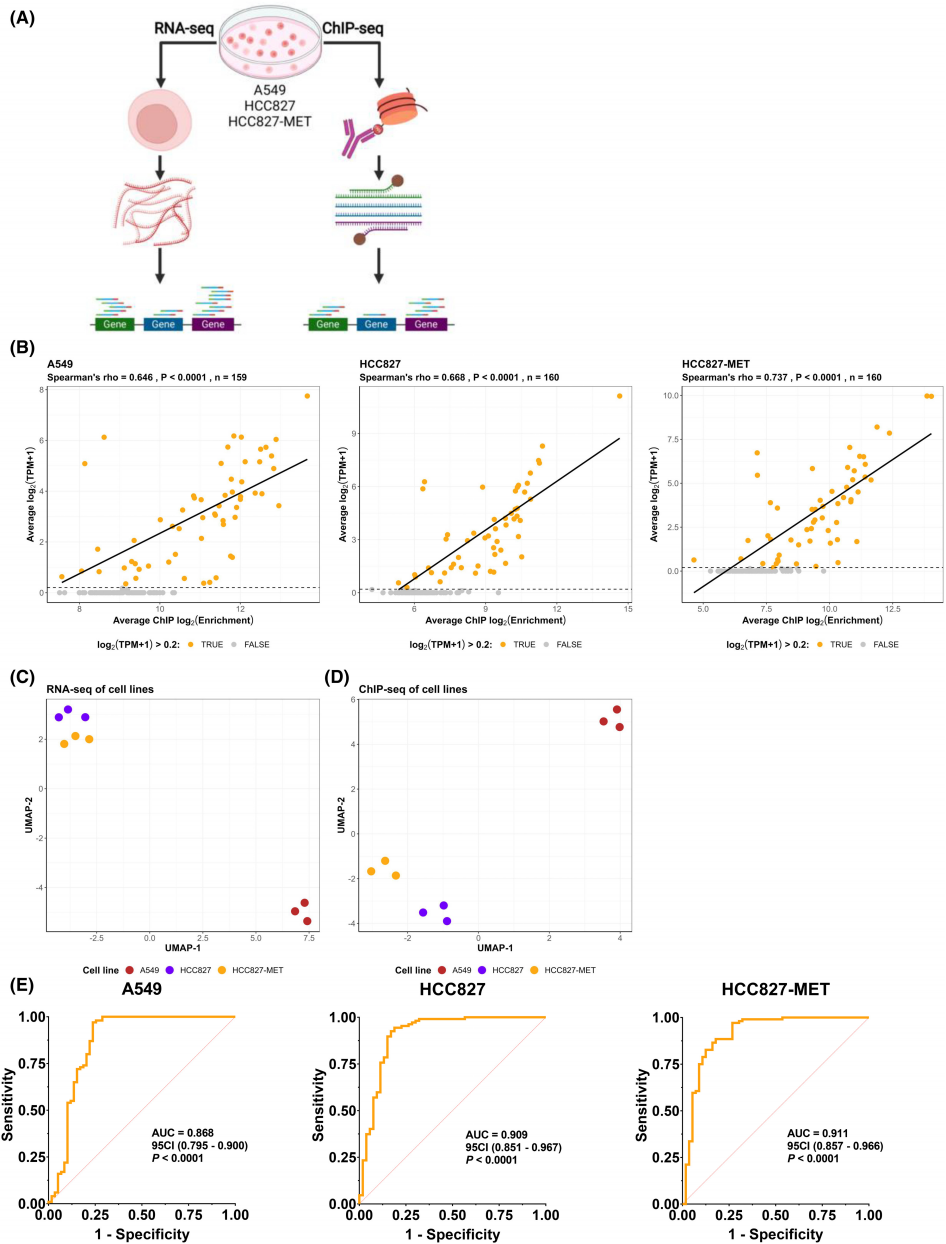
Comparison between chromatin immunoprecipitation sequencing (ChIP‐seq) and RNA‐seq in cell lines. (A) Experimental set‐up. Left: RNA was purified from cell cultures and subjected to paired‐end sequencing. Right: H3K36me3 ChIP was performed on the same cell culture as RNA‐seq. Following H3K36me3 ChIP enrichment the isolated DNA was subjected to Cancer personalised profiling by deep sequencing (CAPP‐seq). Created with www.biorender.com. (B) Correlation between mRNA expression levels and H3K36me3 ChIP enrichment in A549, HCC827, and HCC827‐MET cells. *P*‐values are calculated using the algorithm AS 89. (C) Uniform Manifold Approximation and Projection (UMAP) of triplicate RNA‐seq experiments of A549, HCC827, and HCC827‐MET cell lines. The UMAP is based on transcripts per million (TPM) values of 53 585 transcripts. (D) UMAP of triplicate ChIP‐seq for A549, HCC827, and HCC827‐MET. The UMAP is based on enrichment values of 197 genes in the AVENIO gene panel. (E) Receiver operating characteristic (ROC) analysis of the ability for H3K36me3 ChIP to determine if a gene is active or inactive where the area under the curve (AUC) is indicated for each cell line. The two‐tailed *P*‐values are calculated using a *z*‐test where the *z*‐ratio is calculated as (AUC‐0.50)/SE.

In order to demonstrate the correlation between mRNA expression levels and H3K36me3 ChIP enrichment, RNA‐seq and H3K36me3 ChIP‐seq was performed for the three separate cell lines, HCC827, HCC827‐MET and A549 (Fig. 1A). A549 and HCC827 are two separate cell lines with different oncogenic drivers (KRAS for A549 and EGFR for HCC827). HCC827 is the parental strain from which HCC827‐MET is derived by escalating erlotinib concentrations, whereby the cell line has become resistant to erlotinib [35]. In this cell line, the erlotinib resistance is caused by a *MET* amplification enabling a bypass mechanism for EGFR‐dependency. Previous results have demonstrated that increased H3K36me3 enrichment is a result of increased gene expression [45, 46] and that H3K36me3 is primarily located in the 3′ end of actively transcribed genes [33, 47]. This results in low H3K36me3 deposition close to the transcription start site (TSS) [48]. Unfortunately, some genes in the AVENIO surveillance panel are only captured in the beginning of the gene (Fig. S1). Based on this, we have excluded genes solely sequenced in the first 25% from the TSS from further ChIP‐seq analysis in order to avoid false‐negative results (Table S6, Fig. S2).

We hypothesised that the level of H3K36me3 ChIP enrichment would correlate with mRNA expression levels. In Fig. 1B, the normalised average mRNA expression levels are plotted relative to the average H3K36me3 ChIP enrichment for A548, HCC827, and HCC827‐MET cells. A correlation between mRNA expression and H3K36me3 ChIP is observed for all three cell lines (A549: *r* = 0.65, *P* < 0.0001, HCC827: *r* = 0.67, *P* < 0.0001, HCC827‐MET: *r* = 0.74, *P* < 0.0001), similar to previous reports of H3K36me3 enrichment compared with mRNA expression [45, 46].

Uniform Manifold Approximation and Projection (UMAP) of triplicate RNA‐seq experiments revealed clear clustering of the different cell types (Fig. 1C). The UMAP classified HCC827‐MET cells in close proximity to HCC827 cells compared with A549 which is in accordance with HCC827‐MET deriving from HCC827 cells. Similar clustering was observed with UMAP of H3K36me3 ChIP enrichment results (Fig. 1D). Again, The HCC827‐MET and HCC827 cells were in close proximity, whereas the A549 cells localised further away from HCC827 and HCC827‐MET cells.

We hypothesised that active genes could be discriminated from inactive genes based on H3K36me3 enrichment. This ability was investigated using receiver operating characteristic (ROC) analysis. Active genes were defined to have a log_2_(TPM + 1) > 0.2. Figure 1E illustrates how H3K36me3 ChIP‐seq can successfully differentiate between active and inactive genes with an area under the curve (AUC) in all three cell types between 0.87 and 0.91. At specificity cut‐off of 0.75, the sensitivity estimates are 0.97, 0.95, 0.88 in A549, HCC827 and HCC827‐MET, respectively. Collectively, the results in Fig. 1 demonstrate that H3K36me3 ChIP enrichment correlates with mRNA expression levels and can successfully differentiate between active and inactive genes.

### 3.2 H3K36me3 ChIP can identify genes with different mRNA expression levels

**Fig. 2:**
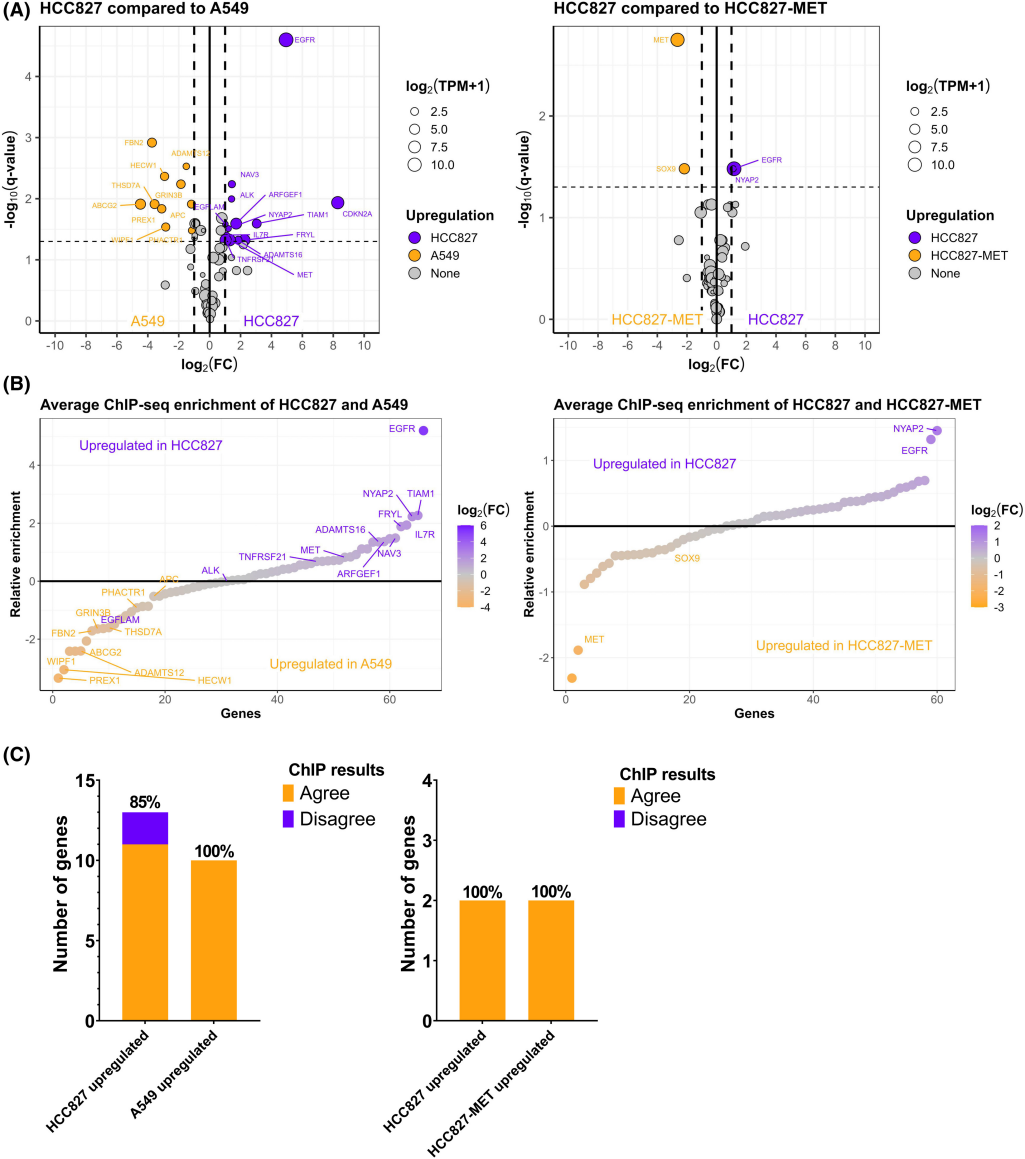
Chromatin immunoprecipitation sequencing (ChIP‐seq) can detect different gene expression levels between cell lines. (A) Volcano plots representing RNA‐seq of HCC827 compared to A549 and HCC827 compared to HCC827‐MET (*n* = 3). False discovery rate (FDR) adjusted log_10_(*q‐*values) are plotted relative to the average log_2_(FC). Genes with an absolute log_2_(FC) > 1 and *q‐*value < 0.05 are labeled. (B) log_2_ average ChIP‐seq enrichment of HCC827‐MET and A549 compared to HCC827 separately (*n* = 3). Labels indicate differentially expressed genes based on RNA‐seq upregulated in HCC827 (purple), A549 (yellow, *left*) or HCC827‐MET (yellow, *right*). (C) Analysis of the agreement between H3K36me3 ChIP‐seq enrichment and RNA‐seq results. H3K36me3 ChIP‐seq is designated to agree with RNA‐seq if a gene displays RNA log_2_(FC) > 1, *q*‐value < 0.05 and H3K36me3 ChIP enrichment.

To investigate whether genes with different mRNA expression levels in the analysed cell lines also could be identified with H3K36me3 ChIP, we compared RNA‐seq and ChIP‐seq results. First, we identified differentially expressed genes (defined as average log_2_(FC) > 1 and *q*‐value < 0.05) between HCC827 and A549 as well as between HCC827 and HCC827‐MET (Fig. 2A). More genes were differently expressed between HCC827 and A549 compared with HCC827 and HCC827‐MET. This is also expected given that A549 and HCC827 are two separate cell lines, whereas HCC827 is the parental strain of HCC827‐MET. HCC827 display increased *EGFR* RNA expression compared with both A549 and HCC827‐MET. Furthermore, HCC827‐MET display increased *MET* expression compared to HCC827, in concordance with the *MET* amplification in HCC827‐MET.

Concordant gene expression profiles were discovered with ChIP‐seq, exemplified with increased *EGFR* H3K36me3 ChIP enrichment in HCC827 compared with HCC827‐MET and A549 (Fig. 2B). A549 has a *CDKN2A* deletion and *CDKN2A* is therefore excluded from Fig. 2B *left*. Similarly, HCC827‐MET displayed increased *MET* H3K36me3 ChIP enrichment compared to HCC827 cells. H3K36me3 ChIP‐tracks of individual genes reveal increased read depth for individual cell lines that correspondingly have more mRNA expression (Fig. S3). By defining differentially expressed genes as log_2_(FC) > 1 or log_2_(FC) < −1 (either twofold increase or decrease) with *q‐*value < 0.05 based on mRNA expression levels, we estimated the sensitivity of ChIP‐seq to detect these genes. In total, 13 genes were upregulated in HCC827 cells compared with A549 cells and of these genes, 11 (0.85 sensitivity) were more enriched in HCC827 by H3K36me3 ChIP‐seq (Fig. 2C *left*). Ten genes have increased mRNA expression levels in A549 cells and all of these (1.00 sensitivity) were more enriched in A549 cells compared with HCC827 by H3K36me3 ChIP‐seq. For HCC827 compared with HCC827‐MET, four genes were differentially expressed with two genes upregulated in HCC827 and HCC827‐MET, respectively. Correspondingly, the four genes demonstrated increased H3K36me3 enrichment in the expected cell line (1.00 sensitivity, Fig. 2C *right*). Combined, H3K36me3 ChIP‐seq was found to be able to detect gene expression differences between cell lines (Fisher's exact test, *P* < 0.0001).

### 3.3 H3K36me3 cfChIP‐seq reveals genes with different enrichment in NSCLC patients compared with healthy individuals

Blood plasma H3K36me3 cfChIP‐seq was performed in eight NSCLC patients, four SCLC patients and four healthy individuals (Table S4). As expected, healthy individuals had a lower mean cfDNA concentration (3.5 ng·mL^−1^, 95% CI = 2.0–5.1 ng·mL^−1^) compared with cancer patients (36.9 ng·mL^−1^, 95% CI = 14.6–59.3 ng·mL^−1^) [29, 49, 50]. This was also evident in the NGS output where the number of deduplicated reads correlated with the amount of cfDNA used for cfChIP‐seq (Spearman's rho = 0.90, *P* < 0.0001; Fig. S4).

We compared the H3K36me3 cfChIP‐seq enrichment of genes from the AVENIO panel in healthy individuals with NSCLC patients. In healthy individuals, cfDNA primarily originate from hematopoietic cells [18, 21, 51], and we therefore hypothesise that H3K36me3 cfChIP‐seq enrichment in healthy individuals reflects the gene expression pattern in PBMCs. We compared RNA‐seq data from NSCLC tumours (GSE179879, *n* = 18) and PBMC RNA‐seq data (GSE107011, *n* = 13) from two previously published data sets [14, 37]. The differential mRNA expression analysis of genes included in the AVENIO surveillance panel demonstrated that *MET*, *EGFR*, *SLPI*, and *TNFRSF21* had the highest difference in log_2_(TPM + 1) between NSCLC tumours and PBMCs (Fig. S5). Interestingly, these four genes were all more enriched in NSCLC H3K36me3 cfChIP‐seq compared with H3K36me3 cfChIP‐seq, using plasma from healthy individuals (Fig. S5, Table S7). That H3K36me3 cfChIP‐seq shows more enrichment of these genes in NSCLC patients compared to healthy individuals indicate that H3K36me3 cfChIP‐seq is a surrogate measure for tumour mRNA expression levels.

### 3.4 Lung cancer mutated genes are enriched in H3K36me3 cfChIP‐seq

**Fig. 3:**
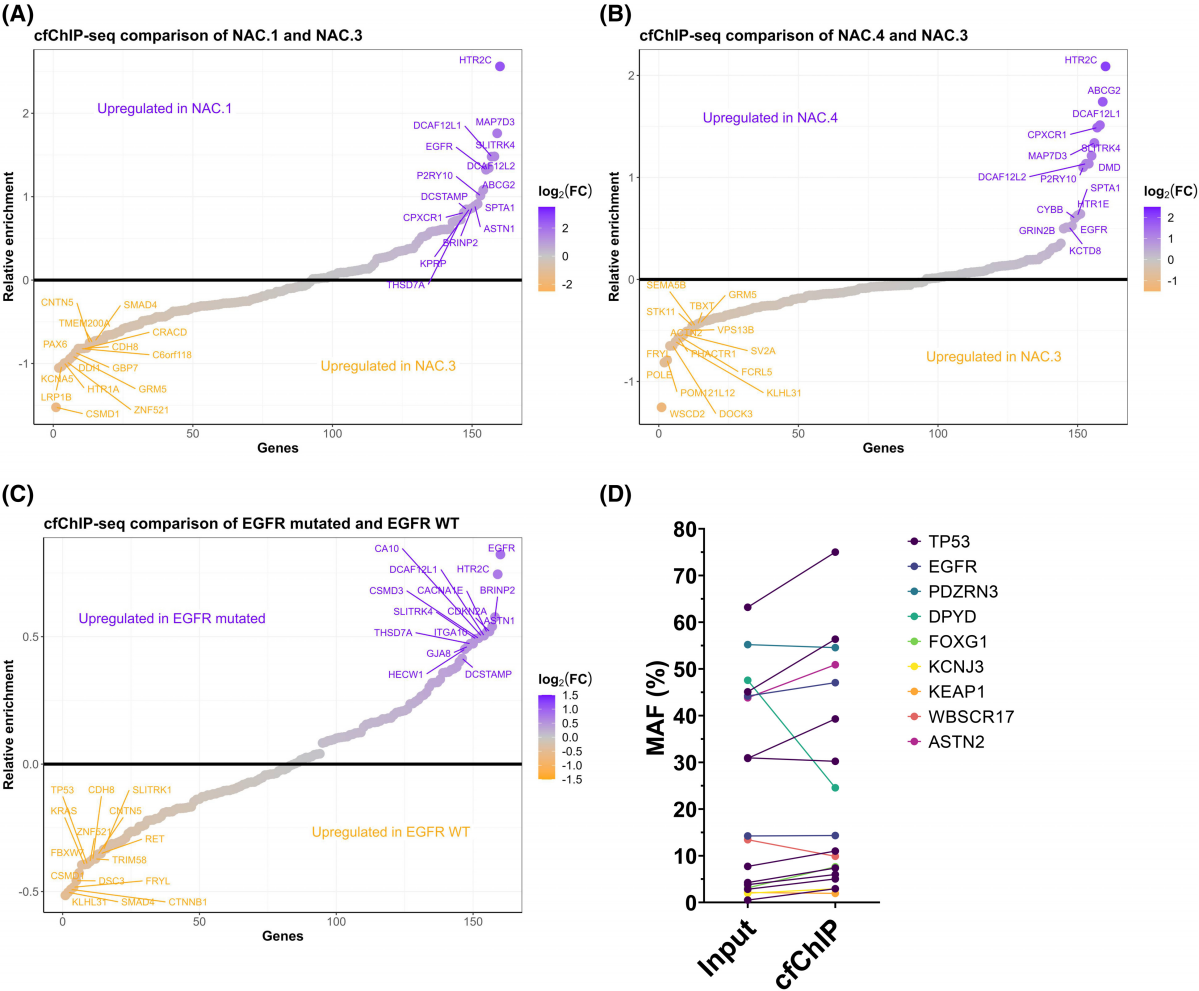
Mutated genes display different cell‐free chromatin immunoprecipitation (cfChIP) enrichment. (A) Relative enrichment of cfChIP in NAC.1 compared to NAC.3. Top 15 most differently enriched genes are displayed for each patient. (B) Relative enrichment of cfChIP in NAC.3 compared to NAC.4. Top 15 most differently enriched genes are displayed for each patient. (C) Average relative enrichment of epidermal growth factor receptor (EGFR) mutated non‐small cell lung cancer (NSCLC) patients (*n* = 2) compared to EGFR WT NSCLC patients (*n* = 6). Top 15 most differently enriched genes are displayed for each group. (D) The mutational allele fraction (MAF) of all mutations identified in both cfChIP and input samples. The MAF is plotted in paired input and cfChIP samples respectively.

We hypothesise that the background H3K36me3 cfChIP enrichment observed in healthy individuals also must be present in cancer patients. To eliminate such background H3K36me3 cfChIP enrichment for the cancer patients, we subtracted the average gene cfChIP enrichment determined for healthy individuals from the cfChIP enrichment determined for each patient (for more details, see Section 2). We have previously shown that mutated *EGFR* fragments are enriched in H3K36me3 cfChIP samples for patients harboring the *EGFR‐L858R* mutation [20]. Based on this, we hypothesised that patients with *EGFR* mutations would have increased *EGFR* H3K36me3 cfChIP enrichment compared to patients without *EGFR* mutations. From the tissue biopsy, an *EGFR* exon 20 insertion was detected in NAC.1. Comparison of H3K36me3 cfChIP between NAC.1 and NAC.3 revealed higher *EGFR* enrichment in NAC.1 (Fig. 3A). Sequencing of the cfChIP input plasma sample for NAC.4 revealed an *EGFR‐L858R* mutation (Allele fraction = 14.2%) despite this patient *in prior* was determined to be free of *EGFR* mutations. The mutation was verified with ddPCR using an *EGFR‐L858R* specific assay as described previously [20] (Fig. S6). Similar to NAC.1, NAC.4 displayed increased *EGFR* H3K36me3 cfChIP enrichment compared with NAC.3 (Fig. 3B). Additional comparisons of H3K36me3 cfChIP enrichments between NSCLC adenocarcinoma patients are displayed in Fig. S7. Both patients with *EGFR* mutations have increased H3K36me3 cfChIP *EGFR* enrichment compared to the non‐*EGFR* mutated patients. However, it should be noted that for NAC.4 compared with NAC.2 *EGFR* is the 28^th^ most enriched gene out of 161. Comparing the average enrichment of the two *EGFR* mutated patients with the remaining *EGFR‐WT* NSCLC patients (*n* = 6) revealed that *EGFR* is the most enriched gene in *EGFR*‐mutated patients (Fig. 3C; Table S8). Interestingly, the *EGFR‐WT* patients now showed increased relative *KRAS* enrichment compared with *EGFR*‐mutated NSCLC patients.

To further address the notion that mutated gene fragments are more enriched because they are more expressed than the WT allele [20], we investigated the allele fraction of mutated genes in input and H3K36me3 cfChIP samples. The mutational allele fraction (MAF) of all mutations identified in both input and cfChIP‐seq samples are displayed in Fig. 3D. Seven of nine genes demonstrate increased MAF in cfChIP samples, whereas *DPYD* and *WBSCR17* have lower MAF in cfChIP sample indicating that the mutated allele is less expressed than the WT alleles. Mutant TP53, found in nine of 12 patients (75%), was the most frequent somatic variant in all the samples. The MAF of *TP53* was significantly higher in cfChIP samples (mean difference: 4.89%, 95% CI = 1.45–8.32%, paired *t*‐test: *P* = 0.0112) which could illustrate tumour‐specific silencing of WT allele relative to mutant TP53 expression.

### 3.5 Blood plasma from NSCLC and SCLC patients display different H3K36me3 cfChIP‐seq profiles

**Fig. 4:**
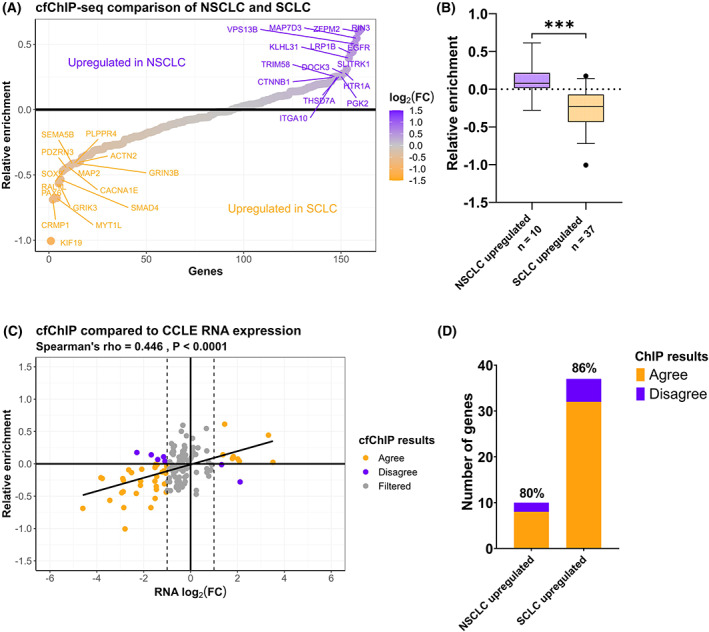
Cell‐free chromatin immunoprecipitation sequencing (cfChIP‐seq) of non‐small cell lung cancer (NSCLC) and small cell lung cancer (SCLC) samples. (A) Average relative H3K36me3 cfChIP‐seq enrichment in NSCLC (*n* = 8) compared to SCLC (*n* = 4) patient blood samples. The top 15 most enriched genes in each group are labeled. (B) The relative enrichment of differentially expressed genes in NSCLC (*n* = 10) and SCLC (*n* = 37) cell lines. *** unpaired *t*‐test, *P* < 0.0001. The error bars indicate 5–95 percentile. (C) Correlation between the relative enrichment in NSCLC/SCLC cfChIP samples and the log_2_(FC) RNA levels between NSCLC and SCLC cell lines. (D) Analysis of agreement between H3K36me3 cfChIP and Cancer Cell Line Encyclopedia (CCLE) mRNA expression data. H3K36me3 cfChIP‐seq is designated to agree with CCLE if a gene displays RNA log_2_(FC) > 1, *q*‐value < 0.05 and H3K36me3 ChIP enrichment.

In order to detect differences in gene expression between NSCLC and SCLC patients, H3K36me3 cfChIP‐seq from eight NSCLC patients was compared with four SCLC patients (Table S1). Figure 4A displays the differential gene enrichment in the two types of tumours following H3K36me3 cfChIP‐seq (Table S9). In order to verify that the genes found to be more H3K36me3 cfChIP enriched in either NSCLC and SCLC plasma samples also represents differences in tumour mRNA expression levels between the two subtypes, SCLC and NSCLC cell line mRNA expression data from the DepMap database was used [36] (Table S2). Some of the genes that were more H3K36me3 cfChIP enriched in NSCLC samples (Relative enrichment > 0), including *EGFR* and *RIN3* were also identified as differentially expressed between SCLC and NSCLC cell lines (Fig. S8). Similarly, genes enriched in SCLC samples (Relative enrichment < 0), including *KIF19*, *CRMP1*, *MYT1L*, *SMAD4*, *PDZRN3*, *GRIK3* and *MAP2* were also more expressed in SCLC cell lines (Fig. S8). These results indicate that differently H3K36me3 cfChIP enriched genes between NSCLC and SCLC plasma samples represent genes which are de facto differentially expressed in the two types of tumours.

We next identified differentially expressed genes between SCLC and NSCLC cell lines (log_2_(FC) > 1 and *q*‐value < 0.05). In Fig. 4B, the relative H3K36me3 cfChIP enrichment in NSCLC samples compared with SCLC samples for genes with increased mRNA expression in either NSCLC or SCLC cell lines is plotted. As expected, the relative H3K36me3 cfChIP enrichment for NSCLC upregulated genes is significantly different from the relative enrichment for genes upregulated in SCLC cell lines (unpaired *t*‐test, *P <* 0.0001). Genes upregulated in NSCLC cell lines had a higher relative H3K36me3 cfChIP enrichment corresponding to high expression in NSCLC blood plasma. Similarly, genes upregulated in SCLC cell lines had a lower relative H3K36me3 cfChIP enrichment, when comparing the NSCLC/SCLC ratio, corresponding to a higher expression in SCLC blood plasma. A positive correlation was observed between the relative enrichment of cfChIP‐seq and the log_2_(FC) for mRNA levels in cell lines between NSCLC and SCLC samples (Fig. 4C, Spearman's rho = 0.45, *P* < 0.0001). By defining differentially expressed genes as having log_2_(FC) > 1 or log_2_(FC) < −1 with *q*‐value < 0.05 in cell lines, we evaluated the ability of H3K36me3 cfChIP‐seq to detect these genes. For NSCLC, 10 genes were upregulated, of which eight (0.80 sensitivity) were H3K36me3 cfChIP enriched in NSCLC samples compared with SCLC samples (Fig. 4D). For SCLC, 37 genes were upregulated and of these 32 (0.86 sensitivity) were H3K36me3 cfChIP enriched in SCLC samples compared to NSCLC samples. A Fisher's exact test of these results demonstrate that H3K36me3 cfChIP‐seq can successfully detect differentially expressed genes between NSCLC and SCLC samples (*P* < 0.0001). The sensitivity of 0.80 and 0.86 for NSCLC and SCLC respectively are similar to results obtained from H3K36me3 ChIP‐seq compared to RNA‐seq of NSCLC cell lines (Fig. 2C) revealing that H3K36me3 cfChIP‐seq can detect differential gene expression between tumour subtypes.

## 4 Discussion

In this study, we demonstrate that H3K36me3 ChIP enrichment can successfully differentiate between active and inactive genes. This is shown for both NSCLC and SCLC patients and in corresponding cell lines. ChIP‐seq enrichment corresponds to mRNA expression levels in cell lines that indicates that H3K36me3 enrichment analyses can work as surrogate for mRNA expression analyses. We extended the use of these observations by performing cfChIP‐seq on plasma samples in order to quantify gene expression in tumours from NSCLC and SCLC patients. Using plasma samples, patients with known tumour mutations revealed increased H3K36me3 cfChIP enrichment for mutated relative to WT alleles for seven of nine genes presented with mutations, including the tumour supressor gene, *TP53*, and the oncogene, *EGFR*. The reduced enrichment following H3K36me3 cfChIP of *TP53* WT relative to mutated alleles is in accordance with transcriptional silencing of the *TP53* WT locus being a cancer driving mechanism acting in parallel with acquirement of loss‐of‐function mutations acting at the post‐transcriptional level. Two of nine mutated genes revealed reduced H3K36me3 enrichment indicating low transcriptional activity in the tumour. Future cfChIP studies targeting closed chromatin markers such as H3K27me3 and H3K9me3 [52] could help to identify inactive genes that can help elucidate the relevance of co‐occurring mutations. Furthermore, we were able to detect genes with different H3K36me3 cfChIP enrichment in NSCLC and SCLC plasma samples. These genes corresponded to genes with differential mRNA expression in NSCLC and SCLC cell lines in support of the utility of H3K36me3 cfChIP‐seq enrichment analysis to determine tumour‐specific mRNA expression levels.

In healthy individuals, cfDNA primarily originate from blood cells [53]. Thus, it shall be acknowledged that cfDNA from cancer patients also contain circulating DNA beyond the ctDNA [18, 24]. In order to increase the sensitivity for tumour‐specific gene enrichment by H3K36me3 cfChIP‐seq, the average gene read counts from four healthy individuals were subtracted from the H3K36me3 cfChIP‐seq gene read counts in the cancer patients. This reduces the amount of background H3K36me3 cfChIP enrichment in cancer samples, for example contributed by leukocyte cfDNA.

In this study, we performed NGS of H3K36me3 ChIP material using a hybridisation capture method [43, 44], allowing the quantification for 197 genes. This method is ultrasensitive for detection of somatic variants in plasma because of the high coverage in the captured fragments. This is also important in cfChIP‐seq experiments because it allows the capture and sequencing of many fragments for each gene, increasing the likelihood of discovering tumour gene expression. Moreover, the high coverage enables the possibility of detecting gene expression differences of single genes, as demonstrated by increased *EGFR* enrichment in *EGFR* mutated NSCLC patients. This is more difficult with a genome‐wide approaches, where transcriptional programmes of gene sets are more in focus [18]. However, in this study, the confidence intervals of the mean relative gene enrichment between groups does overlap 0 for most genes (Table S7‐S9), which indicates that cfChIP‐seq lacks the power to detect gene expression differences consistently on a single gene level for the genes selected here. Nonetheless, the results in Figs 3 and 4 demonstrate how cfChIP‐seq can detect gene expression patterns in tumours of different molecular and histological subtypes when combining several genes. Using cfChIP‐seq on a larger cohort of NSCLC and SCLC patients could result in more confident estimates of single gene expression profiles between the two tumour types.

Applying a smaller commercial gene panel gives higher coverage at a cheaper cost, than, for example, exome‐seq, and could give more reproducible results than a customised gene panel. However, this study is also limited by the area in which the hybridisation panel captures genes, as the initial 25% from the start site of the genes cannot be precipitated with H3K36me3, resulting in a reduction of the genes included in the analysis (Table S6).

In future, cfChIP‐seq can be used to differentiate between different histological and molecular cancer subtypes based on liquid biopsies. Furthermore, cfChIP‐seq can be used to study tumour biology at disease progression including therapy resistance mechanisms. It is now widely accepted that not all resistance mechanisms are caused by the acquisition of novel mutations but could also be a result of changes in gene expression [13]. This includes epithelial‐mesenchymal transition (EMT) [54] as well as NSCLC to SCLC transformation in EGFR tyrosine kinase inhibitor treated patients [55]. In this study we reveal differences in NSCLC and SCLC gene enrichments representative of tumour gene expression. In the future, serial monitoring of NSCLC patients with cfChIP‐seq could identify the onset of NSCLC to SCLC transformation. Furthermore, the differences of H3K36me3 cfChIP enrichment in *EGFR‐WT* and *EGFR‐mutant* tumours (Fig. 3C) reveal of cfChIP‐seq can be used to study gene expression patterns in NSCLC tumours of different molecular subtypes. In future, this can be used to better understand resistance mechanisms of tumours with distinct genetic drivers.

Several studies have now demonstrated that tumour gene expression can be determined in liquid biopsies based on epigenetic features of cfDNA. cfDNA methylation patterns representing gene expression profiles can be traced back to the tissue of origin [56] and has revealed collateral damage in the tissue surrounding the tumour [57]. Moreover, cfDNA fragmentomics have in recent years been studied extensively in order to determine gene expression in the tissue of origin [22, 23, 24, 25]. These methods utilise changes in fragmentation of cfDNA caused by differences in chromatin structure and transcription factor binding around the TSS reflecting the transcriptional activity of the underlaying gene. These patterns are tissue specific and can therefore be used to estimate origin of the cfDNA. In this study, we have focussed on epigenetically modified nucleosomes associated with active transcription. Recently, Sadeh et al. published a study focussing on H3K36me3 and H3K4me3 cfChIP‐seq [18]. Their study revealed that cfChIP‐seq can detect gene expression profiles related to different diseases including colorectal carcinoma, liver diseases and patients with acute myocardial infarction. Another approach to study tumour gene expression could be to study cell‐free RNA (cfRNA), which include both miRNA and mRNA [58]. Compared with cfDNA and circulating miRNA, mRNA is expected to be less stable in the circulation [59]. However, recently mRNA‐seq and full transcriptome sequencing of cfRNA has been able to determine gene expression patterns in liquid biopsies [60, 61]. Regardless of the approach, the clinical value of being able to determine cell of origin gene expression in liquid biopsies is indisputable and will in the future lead to increased knowledge regarding disease diagnosis, monitoring and progression.

## 5 Conclusion

This study is a proof of principle that H3K36me3 cfChIP‐seq can be a surrogate to quantify tumour gene expression in cancer patients. We demonstrate how RNA expression levels correlate with H3K36me3 enrichment and detect differential enriched genes corresponding to the different tumour cell phenotypes. In the future, cfChIP‐seq of blood plasma or other types of liquid biopsies from cancer patients can help to discover predictive and prognostic biomarkers related to treatment efficacy and resistance caused by changes in tumour gene expression.

## Conflict of interest

The authors declare no conflict of interest.

## Author contributions

CTM, ALN and BSS conceived and designed the study. CTM performed the experiments, analysed the data, and drafted the manuscript. PM and MS contributed with clinical data and patient material. All authors revised and approved the final manuscript.

## Supporting information


**Fig. S1.** Characteristics of the AVENIO surveillance panel.
**Fig. S2.** Average H3K36me3 ChIP‐seq enrichment relative to average mRNA expression log2(TPM+1) levels for included and excluded genes. RNA‐seq and ChIP‐seq was made in triplicates.
**Fig. S3.** ChIP‐seq track concordant to mRNA expression data in A549, HCC827, and HCC827‐MET cells.
**Fig. S4.** Metrics of H3K36me3 cfChIP‐seq samples.
**Fig. S5.** Comparing NSCLC and PBMC RNA‐seq with H3K36me3 cfChIP‐seq in healthy and NSCLC patients.
**Fig. S6.** Droplet digital PCR of NAC.4 and a no template control (NTC).
**Fig. S7.** H3K36me3 cfChIP‐seq enrichment between Adenocarcinoma patients.
**Fig. S8.** CCLE mRNA expression data of 50 SCLC cell lines and 106 NSCLC cell lines.

**Table S1.** Patient characteristics.

**Table S2.** Cells used for differential gene expression analysis.

**Table S3.** RNA‐seq characteristics.

**Table S4.** cfChIP‐seq characteristics.

**Table S5.** ChIP‐seq characteristics.

**Table S6.** Genes sequenced using CAPP‐seq.

**Table S7.** Average enrichment in NSCLC patients (n = 8) and healthy individuals (n = 4).

**Table S8.** Average enrichment in EGFR‐mut (n = 2) and EGFR‐WT (n = 6) NSCLC patients.

**Table S9.** Average enrichment in NSCLC (n = 8) and SCLC (n = 4) patients.

**Table S10.** Raw unique read counts for all ChIP and cfChIP samples.

## Data Availability

This study used RNA‐seq data from two separate GEO datasets (GSE179879 and GSE107011). Raw sequencing data from cell lines used in this study have been deposited in the European Nucleotide Archive (ENA) at EMBL‐EBI under accession number PRJEB56750. This repository contains RNA‐seq as well as ChIP‐seq data of the cell lines used in this study. The gene read counts from each individual and cell lines are available in the Table S10. The remaining data generated for this study is included in the published article as well as the supplementary files.
