# Supplementary material for: Cell‐free chromatin immunoprecipitation can determine tumor gene expression in lung cancer patients
Source: Mol Oncol. 2023 Mar 5;17(5):722–36. doi: 10.1002/1878-0261.13394 (PMC10158780; doi:10.1002/1878-0261.13394)
Supplement: Supplementary file 1 — Fig. S1. Characteristics of the AVENIO surveillance panel. Fig. S2. Average H3K36me3 ChIP‐seq enrichment relative to average mRNA expression log2(TPM+1) levels for included and excluded genes. RNA‐seq and ChIP‐seq was made in triplicates. Fig. S3. ChIP‐seq track concordant to mRNA expression data in A549, HCC827, and HCC827‐MET cells. Fig. S4. Metrics of H3K36me3 cfChIP‐seq samples. Fig. S5. Comparing NSCLC and PBMC RNA‐seq with H3K36me3 cfChIP‐seq in healthy and NSCLC patients. Fig. S6. Droplet digital PCR of NAC.4 and a no template control (NTC). Fig. S7. H3K36me3 cfChIP‐seq enrichment between Adenocarcinoma patients. Fig. S8. CCLE mRNA expression data of 50 SCLC cell lines and 106 NSCLC cell lines. [file MOL2-17-722-s011.pdf]

Supplementary figures

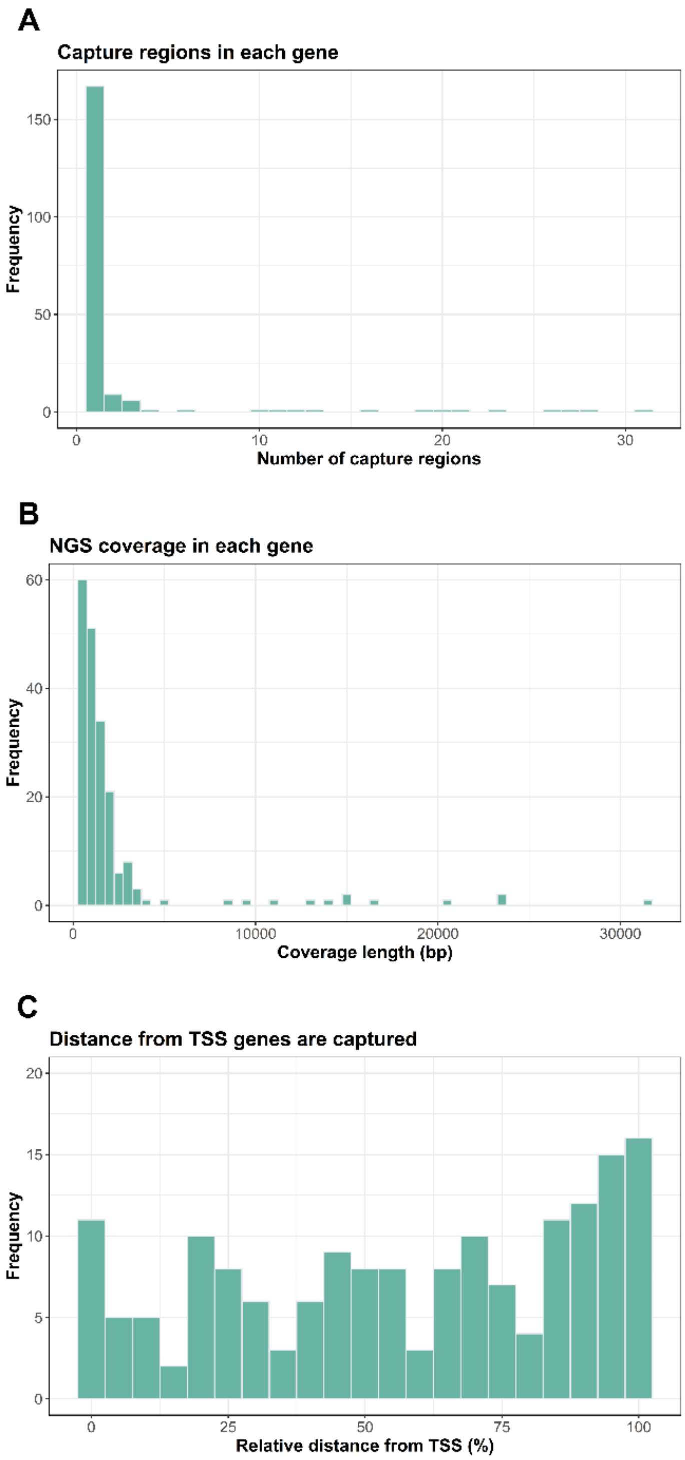

Fig. S1. Characteristics of the AVENIO surveillance panel. (A) Histogram of the number captured regions which are sequenced in each gene. (B) Histogram of the number of bases which are covered in each gene. (C) Histogram of the relative distance from the transcription start site (TSS). Only genes which are captured at a single region are included.

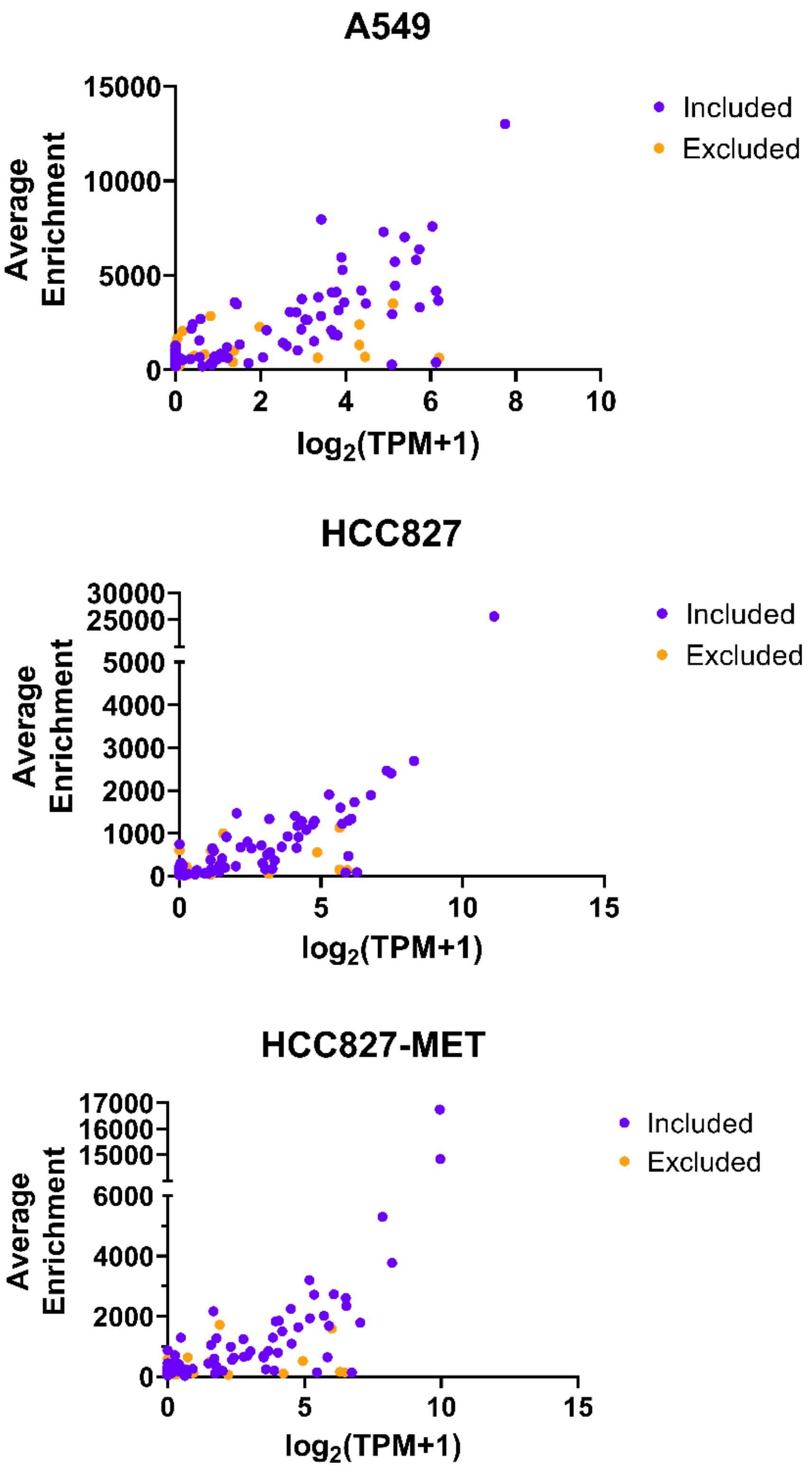

Fig. S2. Average H3K36me3 ChIP-seq enrichment relative to average mRNA expression log_2(TPM+1) levels for included and excluded genes. RNA-seq and ChIP-seq was made in triplicates.

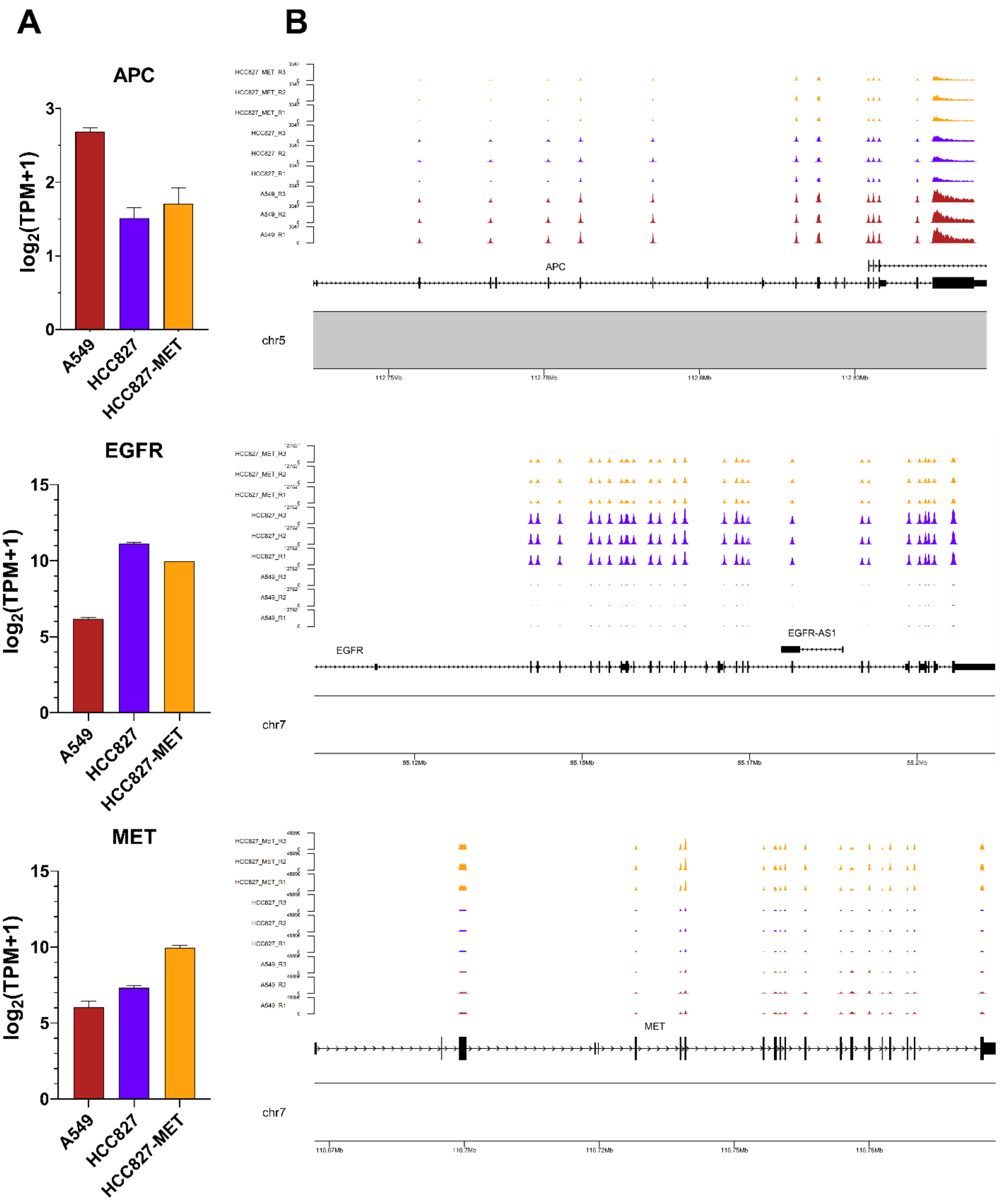

Fig S3. ChIP-seq track concordant to mRNA expression data in A549, HCC827, and HCC827-MET cells. (A) mRNA expression of *APC*, *EGFR*, and *MET* in A549, HCC827, and HCC827-MET cells. The bars and error bars indicate average $\log_2$(TPM+1) and standard deviation (n = 3). (B) H3K36me3 ChIP tracks showing the read depth in *APC*, *EGFR*, and *MET* for A549 (red), HCC827 (purple), and HCC827-MET (yellow) cells.

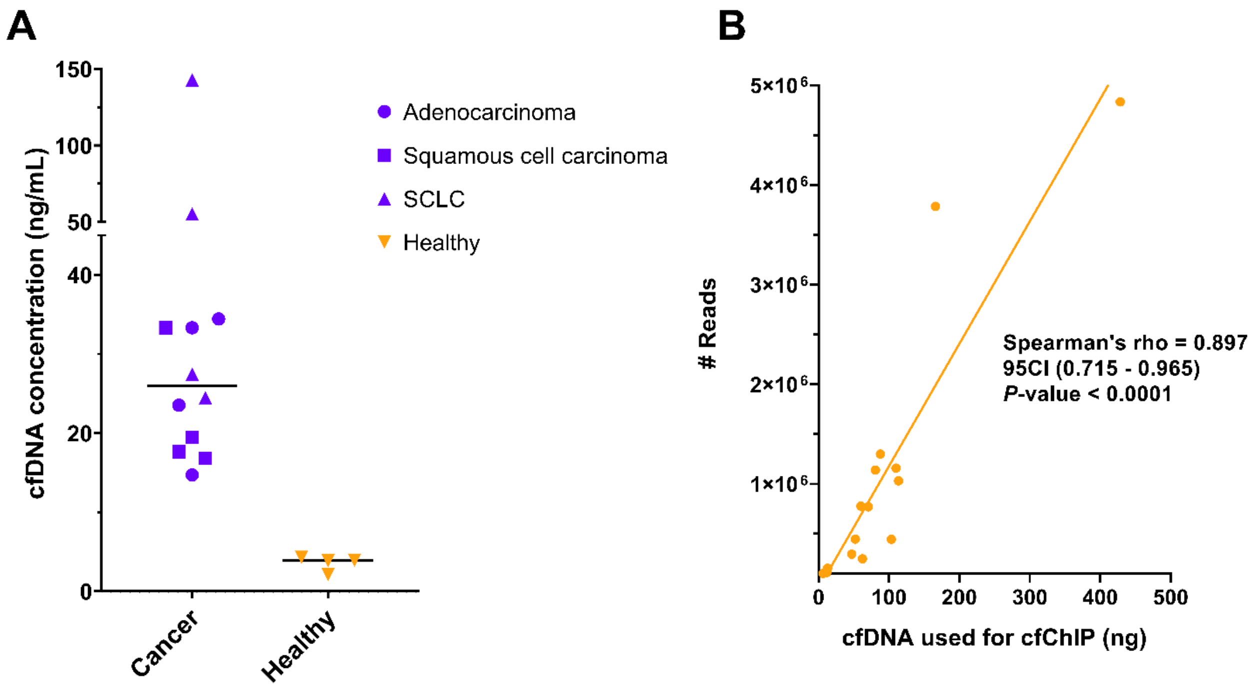

Fig. S4. Metrics of H3K36me3 cfChIP-seq samples. (A) cfDNA concentration in healthy donors (n = 4) and cancer patients (n = 12). (B) Spearman correlation between amount of cfDNA used for cfChIP-seq and number of deduplicated reads generated (n = 16). The *P*-value is calculated using the algorithm AS 89.

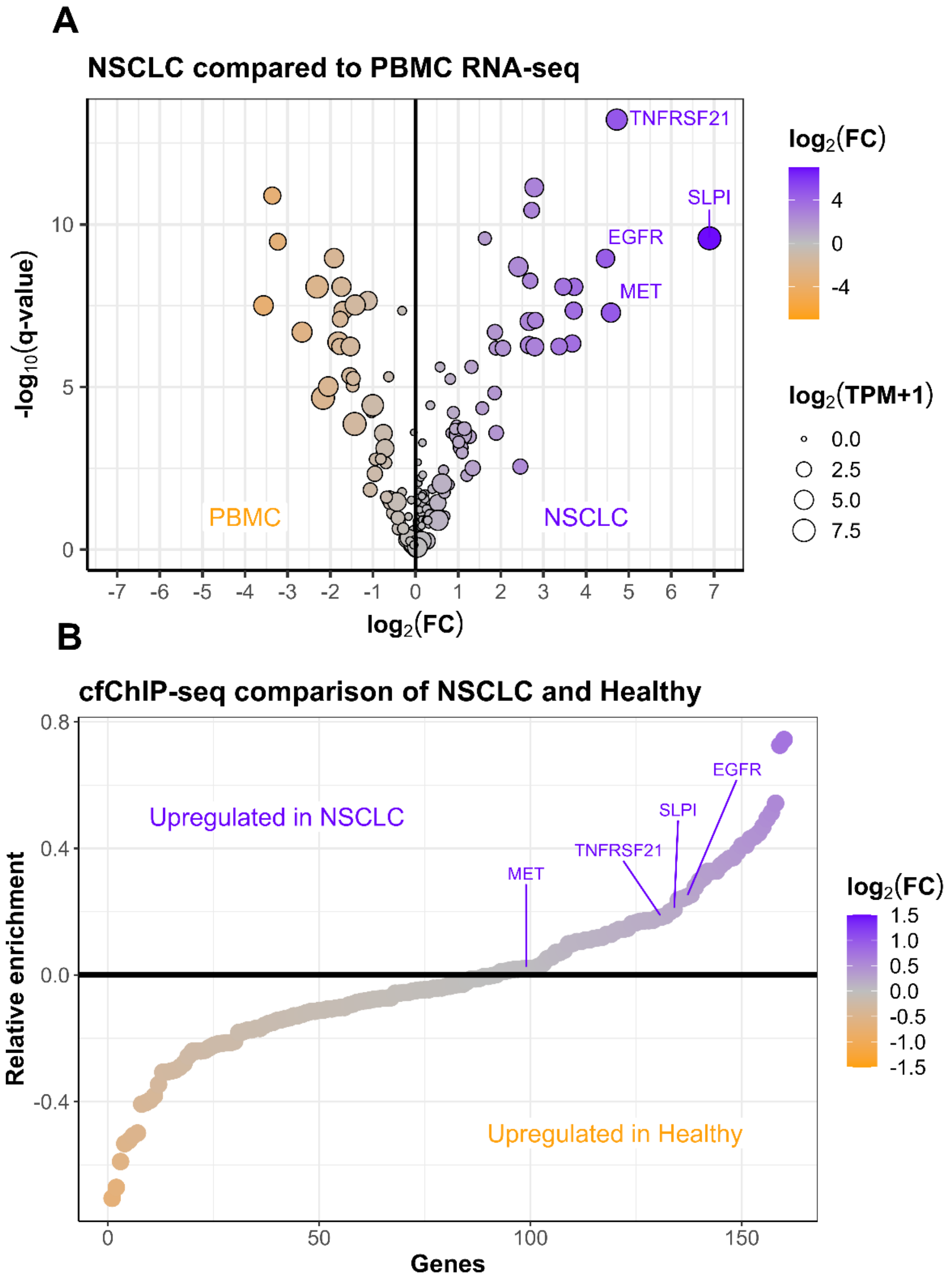

Fig. S5. Comparing NSCLC and PBMC RNA-seq with H3K36me3 cfChIP-seq in healthy and NSCLC patients. (A) NSCLC RNA-seq from GSE179879 (n = 18) [1] was compared to peripheral blood mononuclear cell (PBMC) RNA-seq from GSE107011 (n = 13) [2]. Sizes indicate the average $\log_2$(TPM+1) expression and colors indicate $\log_2$(FC). The q-values are based on unpaired t-tests using FDR for multiple testing adjustment. Genes with $\log_2(FC) > 4$ are labeled. (B) H3K36me3 cfChIP-seq enrichment of plasma from healthy individuals (n = 4) compared to NSCLC patients (n = 8). Colors indicated the average $\log_2$(FC) enrichment between the two groups. The four genes with $\log_2(FC) > 4$ in (A) are labeled.

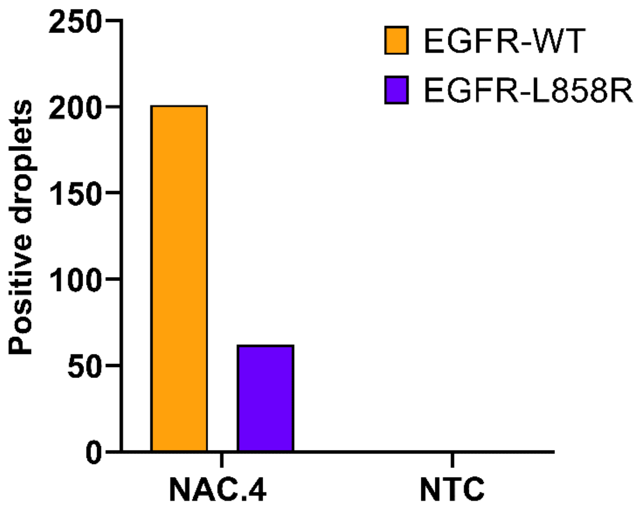

Fig S6. Droplet digital PCR of NAC.4 and a no template control (NTC). The number of positive droplets is displayed in yellow for EGFR-WT and purple for EGFR-L858R. The ddPCR assay is performed as described in [3].

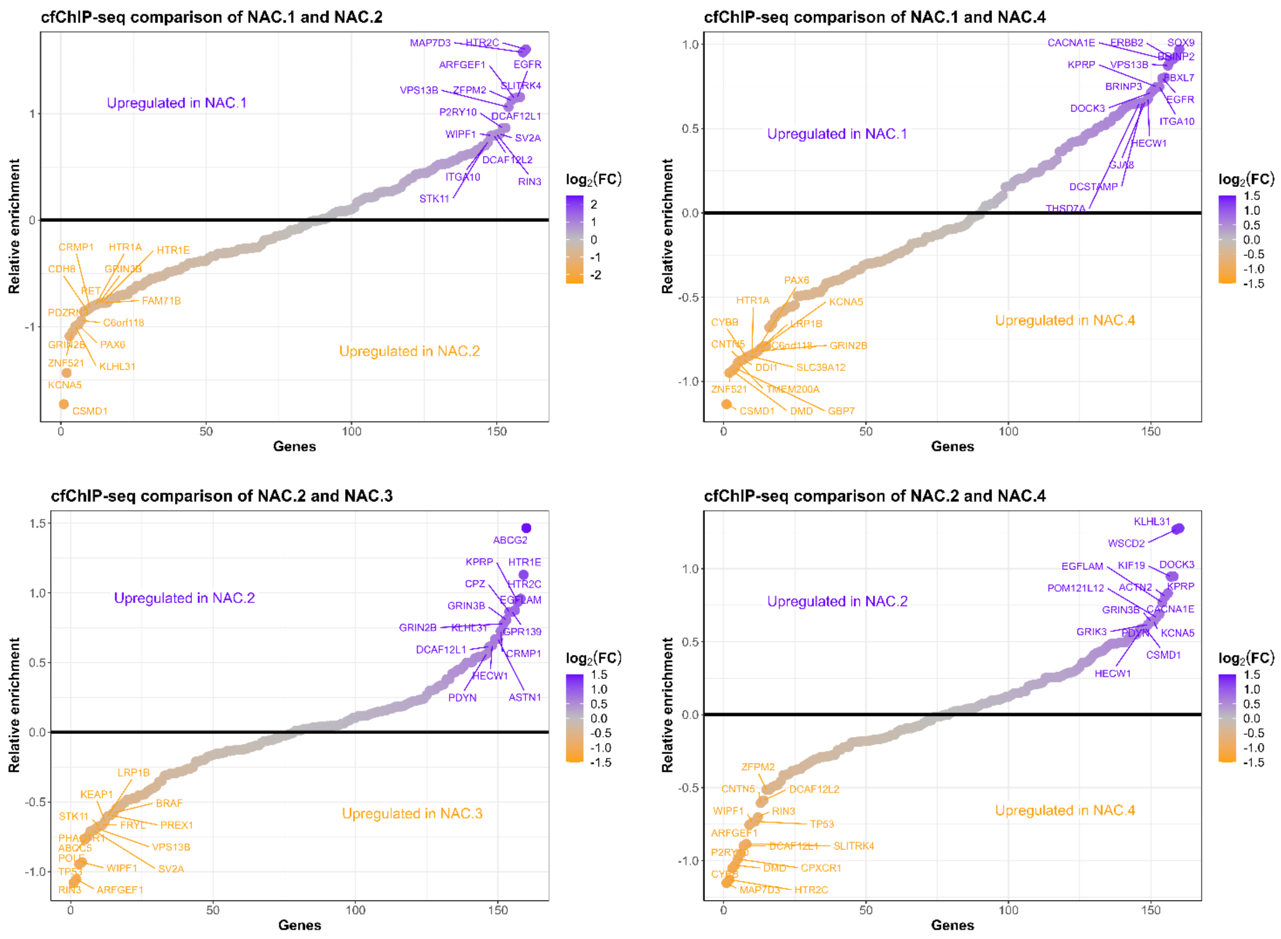

Fig. S7. H3K36me3 cfChIP-seq enrichment between Adenocarcinoma patients.

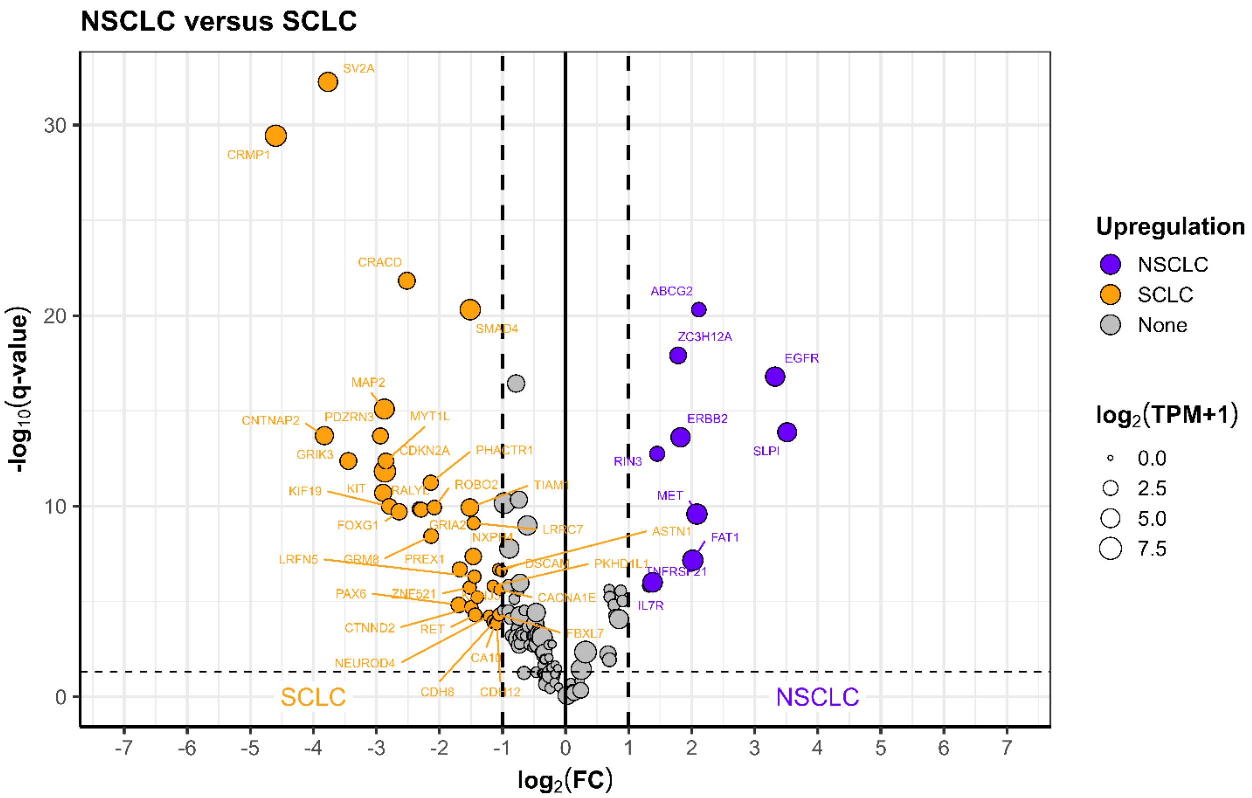

Fig. S8. CCLE mRNA expression data of 50 SCLC cell lines and 106 NSCLC cell lines. Sizes indicate the average log_2(TPM+1) expression and colors indicate log_2(FC). The q-values are based on unpaired t-tests using FDR for multiple testing adjustment. Genes with *q*-value < 0.05 and log_2(FC) > 1 or log_2(FC) < -1 are labeled.

References

1. McCann K, von Witzleben A, Thomas J, Wang C, Wood O, Singh D, et al. Targeting the tumor mutanome for personalized vaccination in a TMB low non-small cell lung cancer. J Immunother Cancer. 2022;10(3).
2. Monaco G, Lee B, Xu W, Mustafah S, Hwang YY, Carré C, et al. RNA-Seq Signatures Normalized by mRNA Abundance Allow Absolute Deconvolution of Human Immune Cell Types. Cell Rep. 2019;26(6):1627-40.e7.
3. Månsson CT, Vad-Nielsen J, Meldgaard P, Nielsen AL, Sorensen BS. EGFR transcription in non-small-cell lung cancer tumours can be revealed in ctDNA by cell-free chromatin immunoprecipitation (cfChIP). Mol Oncol. 2021;15(11):2868-76.
4. Ghandi M, Huang FW, Jané-Valbuena J, Kryukov GV, Lo CC, McDonald ER, 3rd, et al. Next-generation characterization of the Cancer Cell Line Encyclopedia. Nature. 2019;569(7757):503-8.
