# Supplementary material for: Cell‐free chromatin immunoprecipitation can determine tumor gene expression in lung cancer patients
Source: Mol Oncol. 2023 Mar 5;17(5):722–36. doi: 10.1002/1878-0261.13394 (PMC10158780; doi:10.1002/1878-0261.13394)
Supplement: Supplementary file 2 — Table S1. Patient characteristics. [file MOL2-17-722-s004.pdf]

Table S1. Patient characteristics.

Lung cancer patients<br>Total n (%)	12 (100)
Gender	
Female	8 (67)
Male	4 (33)
Age at baseline	
Mean age	69.4
< 69.4	5 (42)
> 69.4	7 (58)
Smoking status	
Former	6 (50)
Current	5 (42)
Never	1 (8)
Tumor type	
NSCLC	8 (67)
SCLC	4 (33)
NSCLC histology	
Adenocarcinoma	4 (50)
Squamous cell carcinoma	4 (50)
Tumor size	
Mean size (mm)	65.8
< 65.8	7 (58)
> 65.8	4 (33)
Unknown	1 (8)
Known mutations	
EGFR-ex20ins	1 (8)
KRAS-G12C	1 (8)
None	10 (84)
