# Supplementary material for: Cell‐free chromatin immunoprecipitation can determine tumor gene expression in lung cancer patients
Source: Mol Oncol. 2023 Mar 5;17(5):722–36. doi: 10.1002/1878-0261.13394 (PMC10158780; doi:10.1002/1878-0261.13394)
Supplement: Supplementary file 3 — Table S2. Cells used for differential gene expression analysis. [file MOL2-17-722-s009.pdf]

Table S2. Cells used for differential gene expression analysis.

NSCLC cells		SCLC cells	
CCLE Name	Depmap ID	CCLE name	Depmap ID
A427_LUNG	ACH-000033	COLO668_LUNG	ACH-000179
A549_LUNG	ACH-000553	CORL24_LUNG	ACH-000297
ABC1_LUNG	ACH-000710	CORL279_LUNG	ACH-000523
CALU1_LUNG	ACH-000737	CORL311_LUNG	ACH-000870
CALU3_LUNG	ACH-000837	CORL47_LUNG	ACH-000129
CORL105_LUNG	ACH-000845	CORL88_LUNG	ACH-000187
DV90_LUNG	ACH-000012	CORL95_LUNG	ACH-000257
EBC1_LUNG	ACH-000021	DMS114_LUNG	ACH-000290
EKVX_LUNG	ACH-000029	DMS153_LUNG	ACH-000292
EPLC272H_LUNG	ACH-000030	DMS273_LUNG	ACH-000298
HARA_LUNG	ACH-000035	DMS454_LUNG	ACH-000355
HCC1171_LUNG	ACH-000062	DMS53_LUNG	ACH-000358
HCC1195_LUNG	ACH-000066	DMS79_LUNG	ACH-000382
HCC15_LUNG	ACH-000109	HCC33_LUNG	ACH-000394
HCC1833_LUNG	ACH-000121	LU135_LUNG	ACH-000399
HCC2108_LUNG	ACH-000150	NCIH1048_LUNG	ACH-000431
HCC2279_LUNG	ACH-000161	NCIH1092_LUNG	ACH-000506
HCC2450_LUNG	ACH-000176	NCIH1105_LUNG	ACH-000508
HCC2814_LUNG	ACH-000261	NCIH1184_LUNG	ACH-000514
HCC2935_LUNG	ACH-000282	NCIH1341_LUNG	ACH-000515
HCC364_LUNG	ACH-000311	NCIH1436_LUNG	ACH-000525
HCC366_LUNG	ACH-000314	NCIH146_LUNG	ACH-000530
HCC4006_LUNG	ACH-000327	NCIH1618_LUNG	ACH-000559
HCC44_LUNG	ACH-000337	NCIH1694_LUNG	ACH-000586
HCC461_LUNG	ACH-000339	NCIH1836_LUNG	ACH-000594
HCC515_LUNG	ACH-000343	NCIH1876_LUNG	ACH-000610
HCC78_LUNG	ACH-000367	NCIH1930_LUNG	ACH-000639
HCC827_LUNG	ACH-000378	NCIH196_LUNG	ACH-000659
HCC827GR5_LUNG	ACH-000379	NCIH1963_LUNG	ACH-000670
HCC95_LUNG	ACH-000390	NCIH2029_LUNG	ACH-000695
HOP62_LUNG	ACH-000392	NCIH2081_LUNG	ACH-000698
KNS62_LUNG	ACH-000395	NCIH209_LUNG	ACH-000703
LC1F_LUNG	ACH-000414	NCIH211_LUNG	ACH-000729
LC1SQSF_LUNG	ACH-000416	NCIH2171_LUNG	ACH-000743
LK2_LUNG	ACH-000442	NCIH2196_LUNG	ACH-000749
LOUNH91_LUNG	ACH-000447	NCIH2227_LUNG	ACH-000752
LUDLU1_LUNG	ACH-000448	NCIH2286_LUNG	ACH-000767
LXF289_LUNG	ACH-000451	NCIH446_LUNG	ACH-000780
MORCPR_LUNG	ACH-000454	NCIH510_LUNG	ACH-000790
NCIH1355_LUNG	ACH-000481	NCIH524_LUNG	ACH-000800
NCIH1373_LUNG	ACH-000482	NCIH526_LUNG	ACH-000803
NCIH1385_LUNG	ACH-000496	NCIH69_LUNG	ACH-000816
NCIH1395_LUNG	ACH-000511	NCIH82_LUNG	ACH-000830

NCIH1435_LUNG	ACH-000521	NCIH841_LUNG	ACH-000844
NCIH1437_LUNG	ACH-000528	NCIH889_LUNG	ACH-000866
NCIH1563_LUNG	ACH-000562	SBC5_LUNG	ACH-000871
NCIH1568_LUNG	ACH-000563	SCLC21H_LUNG	ACH-000890
NCIH1573_LUNG	ACH-000575	SCLC22H_LUNG	ACH-000912
NCIH1623_LUNG	ACH-000578	SHP77_LUNG	ACH-001386
NCIH1648_LUNG	ACH-000585	SW1271_LUNG	ACH-001549
NCIH1650_LUNG	ACH-000587		
NCIH1651_LUNG	ACH-000589		
NCIH1666_LUNG	ACH-000590		
NCIH1693_LUNG	ACH-000628		
NCIH1703_LUNG	ACH-000638		
NCIH1734_LUNG	ACH-000665		
NCIH1755_LUNG	ACH-000666		
NCIH1781_LUNG	ACH-000667		
NCIH1792_LUNG	ACH-000669		
NCIH1793_LUNG	ACH-000675		
NCIH1819_LUNG	ACH-000677		
NCIH1838_LUNG	ACH-000681		
NCIH1869_LUNG	ACH-000690		
NCIH1944_LUNG	ACH-000700		
NCIH1975_LUNG	ACH-000705		
NCIH2009_LUNG	ACH-000706		
NCIH2023_LUNG	ACH-000712		
NCIH2030_LUNG	ACH-000718		
NCIH2073_LUNG	ACH-000731		
NCIH2085_LUNG	ACH-000733		
NCIH2087_LUNG	ACH-000744		
NCIH2122_LUNG	ACH-000747		
NCIH2126_LUNG	ACH-000757		
NCIH2170_LUNG	ACH-000766		
NCIH2228_LUNG	ACH-000769		
NCIH226_LUNG	ACH-000774		
NCIH2291_LUNG	ACH-000779		
NCIH23_LUNG	ACH-000781		
NCIH2342_LUNG	ACH-000785		
NCIH2347_LUNG	ACH-000787		
NCIH2405_LUNG	ACH-000791		
NCIH2882_LUNG	ACH-000840		
NCIH3122_LUNG	ACH-000841		
NCIH322_LUNG	ACH-000843		
NCIH3255_LUNG	ACH-000851		
NCIH358_LUNG	ACH-000852		
NCIH441_LUNG	ACH-000858		
NCIH520_LUNG	ACH-000860		
NCIH522_LUNG	ACH-000861		
NCIH596_LUNG	ACH-000868		
NCIH647_LUNG	ACH-000869		

NCIH650_LUNG	ACH-000872
NCIH838_LUNG	ACH-000875
NCIH854_LUNG	ACH-000878
PC14_LUNG	ACH-000886
PC9_LUNG	ACH-000888
RERFLCAD1_LUNG	ACH-000892
RERFLCAD2_LUNG	ACH-000893
RERFLCAI_LUNG	ACH-000894
RERFLCKJ_LUNG	ACH-000900
RERFLCMS_LUNG	ACH-000916
RERFLCSQ1_LUNG	ACH-000925
SKMES1_LUNG	ACH-000945
SQ1_LUNG	ACH-000951
SW1573_LUNG	ACH-000975
SW900_LUNG	ACH-001113
