# Supplementary material for: Cell‐free chromatin immunoprecipitation can determine tumor gene expression in lung cancer patients
Source: Mol Oncol. 2023 Mar 5;17(5):722–36. doi: 10.1002/1878-0261.13394 (PMC10158780; doi:10.1002/1878-0261.13394)
Supplement: Supplementary file 4 — Table S3. RNA‐seq characteristics. [file MOL2-17-722-s002.pdf]

Table S3. RNA-seq characteristics.

Sample	OD260/280	OD260/230	RINe	28S/18S	Mass (µg)	No. Read pairs	Q20 (%)
A549 rep 1	2.03	1.60	10.00	1.90	2.70	24,129,343	97.88
A549 rep 2	2.04	1.05	10.00	2.00	2.35	24,064,090	97.97
A549 rep 3	2.02	0.43	10.00	1.90	2.67	24,073,554	97.93
HCC827 rep 1	1.90	0.58	10.00	1.90	1.62	24,031,106	97.87
HCC827 rep 2	1.92	0.71	10.00	1.90	1.84	24,100,134	97.83
HCC827 rep 3	2.03	0.40	10.00	2.20	2.00	24,123,822	97.91
HCC827-MET rep 1	1.93	0.87	10.00	2.00	3.13	24,103,898	97.89
HCC827-MET rep 2	1.94	1.08	10.00	2.10	2.13	24,035,890	97.96
HCC827-MET rep 3	2.01	1.46	10.00	1.90	4.32	24,037,596	97.89
