# Supplementary material for: Cell‐free chromatin immunoprecipitation can determine tumor gene expression in lung cancer patients
Source: Mol Oncol. 2023 Mar 5;17(5):722–36. doi: 10.1002/1878-0261.13394 (PMC10158780; doi:10.1002/1878-0261.13394)
Supplement: Supplementary file 5 — Table S4. cfChIP‐seq characteristics. [file MOL2-17-722-s010.pdf]

Table S4. cfChIP-seq characteristics.

Sample ID	Sample type	Histology	Gender	Age	cfDNA concentration (ng/mL)	Plasma for cfChIP (mL)	No. Reads (Deduped)
NAC.1	NSCLC	Adenocarcinoma	F	52.9	34.4	3.3	1,031,221
NAC.2	NSCLC	Adenocarcinoma	M	73.3	33.3	3.3	1,158,106
NAC.3	NSCLC	Adenocarcinoma	M	72.1	14.7	3.2	295,285
NAC.4	NSCLC	Adenocarcinoma	F	66.1	23.5	3.0	769,631
NSC.1	NSCLC	Squamous cell carcinoma	F	78.5	33.3	3.1	444,563
NSC.2	NSCLC	Squamous cell carcinoma	F	79.7	19.5	3.2	248,113
NSC.3	NSCLC	Squamous cell carcinoma	F	73.4	17.6	3.4	777,679
NSC.4	NSCLC	Squamous cell carcinoma	M	81.4	16.8	3.1	445,123
SCC.1	SCLC	NA	F	73.1	142.8	3.0	4,835,016
SCC.2	SCLC	NA	M	65.2	24.5	3.3	1,139,868
SCC.3	SCLC	NA	F	51.7	55.3	3.0	3,785,434
SCC.4	SCLC	NA	F	65.4	27.4	3.2	1,300,234
HC.1	Healthy	NA	M	33.0	2.1	3.0	102,499
HC.2	Healthy	NA	M	49.0	3.9	3.0	111,357
HC.3	Healthy	NA	M	47.0	4.3	3.0	154,167
HC.4	Healthy	NA	F	27.0	3.9	3.0	119,270
