# Supplementary material for: Cell‐free chromatin immunoprecipitation can determine tumor gene expression in lung cancer patients
Source: Mol Oncol. 2023 Mar 5;17(5):722–36. doi: 10.1002/1878-0261.13394 (PMC10158780; doi:10.1002/1878-0261.13394)
Supplement: Supplementary file 6 — Table S5. ChIP‐seq characteristics. [file MOL2-17-722-s003.pdf]

Table S5. ChIP-seq characteristics.

Sample	Mass for NGS (µg)	No. Reads (Deduped)
A549 ChIP rep 1	5.0	3,478,785
A549 ChIP rep 2	6.6	2,872,612
A549 ChIP rep 3	4.3	2,923,622
HCC827 ChIP rep 1	5.3	8,769,589
HCC827 ChIP rep 2	4.0	9,416,096
HCC827 ChIP rep 3	4.2	9,696,190
HCC827-MET ChIP rep 1	2.2	4,398,585
HCC827-MET ChIP rep 2	2.3	5,294,129
HCC827-MET ChIP rep 3	2.3	4,387,125
