# Supplementary material for: Cell‐free chromatin immunoprecipitation can determine tumor gene expression in lung cancer patients
Source: Mol Oncol. 2023 Mar 5;17(5):722–36. doi: 10.1002/1878-0261.13394 (PMC10158780; doi:10.1002/1878-0261.13394)
Supplement: Supplementary file 7 — Table S6. Genes sequenced using CAPP‐seq. [file MOL2-17-722-s005.pdf]

Table S6. Genes sequenced using CAPP-seq.

For each gene the number of regions sequenced and the bp coverage is displayed. Included/excluded determines whether the gene has been excluded from analysis based on the position of the sequencing target region relative to the transcription start site.

SYMBOL	Chr	Gene start	Gene end	Strand	ENSEMBL	No. Regions targeted	Coverage	Included/ excluded
ALK	chr2	29192774	29921586	-	ENSG00000171094	20	16299	Included
APC	chr5	112737885	112846239	+	ENSG00000134982	12	15107	Included
BRAF	chr7	140719337	140924929	-	ENSG00000157764	11	8392	Included
BRCA1	chr17	43044295	43125364	-	ENSG00000012048	23	8392	Included
BRCA2	chr13	32315508	32400268	+	ENSG00000139618	26	8392	Included
DPYD	chr1	97077743	97921034	-	ENSG00000188641	3	2231	Included
EGFR	chr7	55019017	55211628	+	ENSG00000146648	28	31411	Included
ERBB2	chr17	39700064	39728658	+	ENSG00000141736	27	13779	Included
KIT	chr4	54657957	54740715	+	ENSG00000157404	16	9367	Included
KRAS	chr12	25205246	25250929	-	ENSG00000133703	4	3085	Included
MET	chr7	116672196	116798377	+	ENSG00000105976	19	15085	Included
NRAS	chr1	114704469	114716771	-	ENSG00000213281	3	2279	Included
PDGFRA	chr4	54229293	54298245	+	ENSG00000134853	21	13114	Included
RET	chr10	43077069	43130351	+	ENSG00000165731	13	10899	Included
ROS1	chr6	117287353	117425942	-	ENSG00000047936	31	23526	Included
TP53	chr17	7668421	7687490	-	ENSG00000141510	10	5069	Included
UGT1A1	chr2	233760270	233773300	+	ENSG00000241635	1	729	Excluded
ABCC5	chr3	183919934	184017884	-	ENSG00000114770	1	747	Included
ABCG2	chr4	88090150	88231628	-	ENSG00000118777	1	703	Included
ACTN2	chr1	236686499	236764631	+	ENSG00000077522	1	665	Included
ADAMTS12	chr5	33523535	33891990	-	ENSG00000151388	1	708	Included
ADAMTS16	chr5	5140330	5320304	+	ENSG00000145536	1	682	Included
ARFGEF1	chr8	67197658	67343781	-	ENSG00000066777	1	793	Included
ASTN1	chr1	176861067	177164712	-	ENSG00000152092	2	1554	Included
ASTN2	chr9	116425228	117415070	-	ENSG00000148219	1	896	Excluded
AVPR1A	chr12	63142759	63151201	-	ENSG00000166148	1	1364	Excluded
BCHE	chr3	165772904	165837423	-	ENSG00000114200	1	2072	Excluded
BPIFB4	chr20	33081527	33111751	+	ENSG00000186191	1	867	Excluded
BRINP2	chr1	177170958	177282422	+	ENSG00000198797	1	1669	Included
BRINP3	chr1	190097658	190477864	-	ENSG00000162670	1	1619	Included
C6	chr5	41142234	41261438	-	ENSG00000039537	1	737	Included
C6orf118	chr6	165279664	165309605	-	ENSG00000112539	1	1026	Included
CA10	chr17	51630313	52160017	-	ENSG00000154975	1	717	Included
CACNA1E	chr1	181483517	181808084	+	ENSG00000198216	1	875	Included
CDH12	chr5	21750673	22853344	-	ENSG00000154162	1	738	Included
CDH18	chr5	19473048	20575873	-	ENSG00000145526	1	731	Included
CDH8	chr16	61647250	62036438	-	ENSG00000150394	1	818	Included
CDH9	chr5	26880597	27038586	-	ENSG00000113100	3	2564	Included
CDKN2A	chr9	21967752	21994392	-	ENSG00000147889	1	864	Included
CHRM2	chr7	136868669	137020255	+	ENSG00000181072	1	1799	Included
CNTN5	chr11	99020949	100358885	+	ENSG00000149972	2	1543	Included
CNTNAP2	chr7	146116801	148420998	+	ENSG00000174469	3	2221	Included
CPXCR1	chrX	88747225	88754781	+	ENSG00000147183	1	1239	Included
CPZ	chr4	8592660	8619759	+	ENSG00000109625	1	784	Included
CRACD	chr4	56170195	56328625	+	ENSG00000109265	1	3095	Included
CRMP1	chr4	5820783	5888481	-	ENSG00000072832	1	721	Included
CSMD1	chr8	2935361	4994914	-	ENSG00000183117	1	659	Included
CSMD3	chr8	112222928	113436939	-	ENSG00000164796	6	4181	Included
CTNNB1	chr3	41194741	41239949	+	ENSG00000168036	1	715	Included
CTNND2	chr5	10971836	11904446	-	ENSG00000169862	1	762	Included
CYBB	chrX	37780059	37813461	+	ENSG00000165168	1	773	Included
DCAF12L1	chrX	126549383	126552814	-	ENSG00000198889	1	1920	Included
DCAF12L2	chrX	126163499	126166289	-	ENSG00000198354	1	1771	Included
DCAF4L2	chr8	87870747	87874015	-	ENSG00000176566	1	1684	Excluded
DCLK1	chr13	35768652	36131382	-	ENSG00000133083	1	722	Excluded
DCSTAMP	chr8	104339826	104356689	+	ENSG00000164935	1	1477	Included
DDI1	chr11	104036640	104039196	+	ENSG00000170967	1	1715	Included
DLGAP2	chr8	737628	1708476	+	ENSG00000198010	1	875	Included
DMD	chrX	31119222	33211549	-	ENSG00000198947	1	764	Included
DNTTIP1	chr20	45791954	45811418	+	ENSG00000101457	1	679	Excluded
DOCK3	chr3	50674927	51384198	+	ENSG00000088538	1	721	Included
DSC3	chr18	30989365	31042742	-	ENSG00000134762	1	706	Included
DSCAM	chr21	40010999	40847158	-	ENSG00000171587	1	675	Included
EGFLAM	chr5	38258559	38465480	+	ENSG00000164318	1	672	Included
EPHA5	chr4	65319563	65670343	-	ENSG00000145242	1	979	Excluded
EPHA6	chr3	96814586	97749241	+	ENSG00000080224	1	1071	Included

EYS	chr6	63720136	65707214	-	ENSG00000188107	1	1275	Excluded
FAM135B	chr8	138130023	138497261	-	ENSG00000147724	1	2464	Included
FAM151A	chr1	54609181	54623525	-	ENSG00000162391	1	725	Included
FAM71B	chr5	157161846	157166264	-	ENSG00000170613	1	1706	Included
FAT1	chr4	186587794	186723856	-	ENSG00000083857	1	818	Included
FBN2	chr5	128258037	128659185	-	ENSG00000138829	2	1368	Included
FBXL7	chr5	15500180	15939793	+	ENSG00000183580	2	2100	Included
FBXW7	chr4	152320544	152382529	-	ENSG00000109670	2	1342	Included
FCRL5	chr1	157513377	157552515	-	ENSG00000143297	1	742	Included
FOXG1	chr14	28766787	28770277	+	ENSG00000176165	1	1386	Included
FRYL	chr4	48497357	48780279	-	ENSG00000075539	1	790	Included
GALNT17	chr7	71132144	71713599	+	ENSG00000185274	1	666	Included
GBA3	chr4	22692914	22819568	+	ENSG00000249948	1	1122	Included
GBP7	chr1	89131742	89176003	-	ENSG00000213512	1	807	Included
GJA8	chr1	147902795	147909269	+	ENSG00000121634	1	1517	Included
GPR139	chr16	20028239	20073890	-	ENSG00000180269	1	1254	Included
GRIA2	chr4	157220728	157366075	+	ENSG00000120251	1	709	Included
GRIK3	chr1	36795527	37034515	-	ENSG00000163873	1	669	Included
GRIN2B	chr12	13537337	13981602	-	ENSG00000273079	1	965	Included
GRIN3B	chr19	1000419	1009732	+	ENSG00000116032	1	1016	Included
GRM1	chr6	146027782	146437598	+	ENSG00000152822	1	1311	Included
GRM5	chr11	88505039	89066242	-	ENSG00000168959	1	1316	Included
GRM8	chr7	126438615	127253574	-	ENSG00000179603	1	1296	Included
GSX1	chr13	27792483	27794768	+	ENSG00000169840	1	555	Excluded
HACD1	chr10	17589032	17617374	-	ENSG00000165996	1	381	Excluded
HCN1	chr5	45254948	45696380	-	ENSG00000164588	1	1374	Included
HCRTR2	chr6	55106460	55282592	+	ENSG00000137252	1	713	Included
HEBP1	chr12	12974870	13000265	-	ENSG00000013583	1	747	Included
HECW1	chr7	43112647	43566001	+	ENSG00000002746	1	1890	Included
HS3ST4	chr16	25691959	26137685	+	ENSG00000182601	1	1068	Included
HS3ST5	chr6	114055586	114343045	-	ENSG00000249853	1	1426	Included
HTR1A	chr5	63957874	63962445	-	ENSG00000178394	1	1768	Included
HTR1E	chr6	86937528	87016679	+	ENSG00000168830	1	1505	Included
HTR2C	chrX	114584086	114910061	+	ENSG00000147246	1	922	Included
IFI16	chr1	159009892	159055155	+	ENSG00000163565	1	760	Excluded
IL7R	chr5	35856891	35879603	+	ENSG00000168685	1	868	Included
INSL3	chr19	17816512	17821519	-	ENSG00000248099	1	713	Excluded
ITGA10	chr1	145891208	145910050	-	ENSG00000143127	1	699	Included
ITSN1	chr21	33642483	33888608	+	ENSG00000205726	1	722	Included
KCNA5	chr12	5043879	5046788	+	ENSG00000130037	1	1919	Included
KCNB2	chr8	72537225	72938349	+	ENSG00000182674	1	1219	Excluded
KCNC2	chr12	75040078	75209839	-	ENSG00000166006	1	1182	Included
KCNJ3	chr2	154697855	154857873	+	ENSG00000162989	1	997	Included
KCTD8	chr4	44173903	44448809	-	ENSG00000183783	1	992	Included
KEAP1	chr19	10486125	10503356	-	ENSG00000079999	3	3139	Included
KIF17	chr1	20664014	20718007	-	ENSG00000117245	1	483	Excluded
KIF19	chr17	74326226	74355820	+	ENSG00000196169	1	606	Included
KLHL31	chr6	53647916	53665756	-	ENSG00000124743	1	711	Included
KPRP	chr1	152759561	152762052	+	ENSG00000203786	1	2246	Included
LRFN5	chr14	41607570	41904549	+	ENSG00000165379	1	2044	Included
LRP1B	chr2	140231423	142131016	-	ENSG00000168702	1	785	Included
LRRC7	chr1	69568398	70123488	+	ENSG00000033122	1	2207	Included
LRRTM1	chr2	80301878	80304752	-	ENSG00000162951	1	1872	Included
LRRTM4	chr2	76748558	77522368	-	ENSG00000176204	1	2003	Excluded
LTBP4	chr19	40592896	40629818	+	ENSG00000090006	1	633	Excluded
MAP2	chr2	209579609	209734118	+	ENSG00000078018	1	730	Included
MAP7D3	chrX	136213264	136256446	-	ENSG00000129680	1	861	Included
MKRN3	chr15	23565592	23629825	+	ENSG00000179455	1	1927	Excluded
MMP16	chr8	88032011	88327483	-	ENSG00000156103	1	747	Included
MTX1	chr1	155208700	155213831	+	ENSG00000173171	1	561	Excluded
MYH7	chr14	23412740	23435660	-	ENSG00000092054	1	658	Included
MYT1L	chr2	1789124	2331348	-	ENSG00000186487	1	756	Included
NAV3	chr12	77830894	78213010	+	ENSG00000067798	1	1472	Included
NEUROD4	chr12	55019974	55030017	+	ENSG00000123307	1	1476	Included
NFE2L2	chr2	177230308	177264727	-	ENSG00000116044	1	764	Included
NLGN4X	chrX	5890303	6229017	-	ENSG00000146938	1	924	Excluded
NLRP3	chr1	247416156	247449108	+	ENSG00000162711	1	2276	Included
NMUR1	chr2	231523187	231530445	-	ENSG00000171596	1	740	Included
NOL4	chr18	33851100	34223551	-	ENSG00000101746	1	592	Excluded
NPAP1	chr15	24675775	24683393	+	ENSG00000185823	1	3730	Excluded
NR0B1	chrX	30304206	30309390	-	ENSG00000169297	1	1238	Excluded

NRXN1	chr2	49918505	51032561	-	ENSG00000179915	1	1071	Excluded
NXPH4	chr12	57216794	57226449	+	ENSG00000182379	1	893	Included
NYAP2	chr2	225399710	225654018	+	ENSG00000144460	1	1544	Included
OPRD1	chr1	28812170	28871267	+	ENSG00000116329	1	419	Excluded
P2RY10	chrX	78945391	78963727	+	ENSG00000078589	1	1565	Included
PAX6	chr11	31788908	31817961	-	ENSG00000007372	1	648	Included
PCDH15	chr10	53802595	54801291	-	ENSG00000150275	1	1909	Included
PDYN	chr20	1978757	1994285	-	ENSG00000101327	1	914	Included
PDZRN3	chr3	73382431	73624941	-	ENSG00000121440	1	1608	Included
PGK2	chr6	49785660	49787285	-	ENSG00000170950	1	1602	Included
PHACTR1	chr6	12716767	13287837	+	ENSG00000112137	1	635	Included
PIK3CA	chr3	179148357	179240093	+	ENSG00000121879	3	2256	Included
PIK3CG	chr7	106865282	106908980	+	ENSG00000105851	1	2237	Excluded
PKHD1L1	chr8	109362461	109537207	+	ENSG00000205038	1	1315	Included
PLPPR4	chr1	99263953	99309590	+	ENSG00000117600	1	1743	Included
POLE	chr12	132623762	132687342	-	ENSG00000177084	1	758	Included
POM121L12	chr7	53035633	53036925	+	ENSG00000221900	1	1281	Included
PREX1	chr20	48624252	48827999	-	ENSG00000124126	1	720	Included
RALYL	chr8	84183274	84921844	+	ENSG00000184672	1	634	Included
RFX5	chr1	151340640	151347252	-	ENSG00000143390	1	755	Excluded
RIN3	chr14	92513781	92688994	+	ENSG00000100599	1	731	Included
RNASE3	chr14	20891399	20892348	+	ENSG00000169397	1	752	Included
ROBO2	chr3	77039886	77649964	+	ENSG00000185008	1	659	Included
SEMA5B	chr3	122909082	123027878	-	ENSG00000082684	1	633	Included
SLC18A3	chr10	49610310	49612720	+	ENSG00000187714	1	1904	Included
SLC39A12	chr10	17951918	18043285	+	ENSG00000148482	1	625	Included
SLC6A5	chr11	20599608	20659285	+	ENSG00000165970	1	690	Excluded
SLC8A1	chr2	40115031	40512361	-	ENSG00000183023	1	2335	Excluded
SLITRK1	chr13	83877205	83882474	-	ENSG00000178235	1	3395	Excluded
SLITRK4	chrX	143622790	143636101	-	ENSG00000179542	1	2988	Included
SLITRK5	chr13	87672615	87696272	+	ENSG00000165300	1	3124	Included
SLPI	chr20	45252239	45254564	-	ENSG00000124107	1	733	Included
SMAD4	chr18	51029614	51085045	+	ENSG00000141646	1	620	Included
SOX9	chr17	72121020	72126416	+	ENSG00000125398	1	1219	Included
SPTA1	chr1	158610704	158686715	-	ENSG00000163554	1	746	Included
ST6GALNAC3	chr1	76074746	76634603	+	ENSG00000184005	1	807	Included
STK11	chr19	1205778	1228431	+	ENSG00000118046	2	1374	Included
SV2A	chr1	149903318	149917844	-	ENSG00000159164	1	820	Included
TBXT	chr6	166157656	166168619	-	ENSG00000164458	1	723	Included
THSD7A	chr7	11370365	11832198	-	ENSG00000005108	1	1154	Included
TIAM1	chr21	31118416	31558977	-	ENSG00000156299	1	1281	Included
TMEM200A	chr6	130366017	130443067	+	ENSG00000164484	1	1779	Included
TNFRSF21	chr6	47231532	47309905	-	ENSG00000146072	1	865	Included
TNN	chr1	175067833	175148075	+	ENSG00000120332	1	820	Excluded
TNR	chr1	175315194	175743595	-	ENSG00000116147	2	1726	Included
TRHDE	chr12	72272370	72670758	+	ENSG00000072657	1	733	Excluded
TRIM58	chr1	247857187	247880138	+	ENSG00000162722	1	1044	Included
TRPS1	chr8	115408496	115668975	-	ENSG00000104447	1	1659	Excluded
UGT3A2	chr5	36035021	36066882	-	ENSG00000168671	1	678	Included
USH2A	chr1	215622891	216423448	-	ENSG00000042781	2	1802	Included
USP29	chr19	57120043	57131926	+	ENSG00000131864	1	3229	Included
VPS13B	chr8	99013266	99877580	+	ENSG00000132549	1	523	Included
WIPF1	chr2	174562204	174682883	-	ENSG00000115935	1	886	Included
WSCD2	chr12	108129288	108250537	+	ENSG00000075035	1	504	Included
ZC3H12A	chr1	37474580	37484377	+	ENSG00000163874	1	703	Included
ZFPM2	chr8	105318438	105804539	+	ENSG00000169946	1	2960	Included
ZIC1	chr3	147409365	147416719	+	ENSG00000152977	2	3215	Included
ZIC4	chr3	147386046	147406543	-	ENSG00000174963	1	1155	Included
ZNF521	chr18	25061924	25352166	-	ENSG00000198795	1	3713	Included
ZSCAN1	chr19	58034025	58054631	+	ENSG00000152467	1	860	Excluded
