# Supplementary material for: Cell‐free chromatin immunoprecipitation can determine tumor gene expression in lung cancer patients
Source: Mol Oncol. 2023 Mar 5;17(5):722–36. doi: 10.1002/1878-0261.13394 (PMC10158780; doi:10.1002/1878-0261.13394)
Supplement: Supplementary file 8 — Table S7. Average enrichment in NSCLC patients (n = 8) and healthy individuals (n = 4). [file MOL2-17-722-s007.pdf]

Table. S7. Average enrichment in NSCLC patients (n = 8) and healthy individuals (n = 4).
For each gene the Log2FC between NSCLC patients and healthy individuals and 95% confidence interval is calculated

SYMBOL	Average NSCLC enrichment	Average healthy enrichment	Log2FC [95% CI]
ROBO2	1184	1930	-0.7055 [-1.292 - -0.0613]
SLC39A12	2004	3190	-0.6712 [-1.1898 - -0.1734]
RNASE3	1155	1738	-0.5893 [-1.3109 - 0.2433]
MYT1L	1482	2144	-0.5328 [-0.8145 - -0.2761]
KCNA5	2451	3522	-0.5231 [-0.908 - -0.2029]
FAM151A	1394	1981	-0.5075 [-1.122 - 0.1715]
GRIK3	1948	2754	-0.4993 [-0.8988 - -0.1382]
CPZ	1544	2049	-0.4079 [-0.895 - 0.0582]
CRMP1	1304	1724	-0.4031 [-0.7814 - -0.0309]
MMP16	1325	1743	-0.3957 [-0.6474 - -0.1513]
ABCG2	960	1252	-0.3821 [-0.9318 - 0.1928]
WSCD2	1365	1734	-0.3459 [-1.0906 - 0.3407]
CSMD1	1212	1499	-0.3069 [-0.8591 - 0.1568]
C6	1278	1581	-0.3063 [-0.795 - 0.2026]
GRIN2B	1707	2107	-0.3033 [-0.7401 - 0.1323]
ACTN2	1116	1373	-0.2997 [-1.0069 - 0.4866]
FAM71B	2624	3211	-0.2914 [-0.7366 - 0.173]
GPR139	2404	2918	-0.2798 [-0.6654 - 0.1104]
PDYN	2493	2979	-0.2568 [-0.6265 - 0.0945]
SEMA5B	1605	1896	-0.2409 [-0.5308 - 0.0074]
KPRP	3307	3907	-0.2404 [-0.5711 - 0.0289]
PDZRN3	2800	3307	-0.2401 [-0.4849 - -0.0139]
UGT3A2	1684	1986	-0.2379 [-0.5968 - 0.0836]
CNTNAP2	1287	1510	-0.2297 [-0.4729 - -0.009]
KIF19	1377	1607	-0.2223 [-0.7698 - 0.1976]
HTR1E	2613	3039	-0.2179 [-0.5142 - 0.0636]
TBXT	1864	2166	-0.216 [-0.7148 - 0.2747]
ALK	2128	2468	-0.2141 [-0.4636 - 0.0184]
ZIC4	3040	3525	-0.2138 [-0.3665 - -0.067]
MYH7	1732	2003	-0.2097 [-0.9909 - 0.7005]
GRIN3B	2204	2499	-0.1811 [-0.4683 - 0.0842]
ADAMTS16	1670	1891	-0.1792 [-0.7589 - 0.4746]
DSCAM	1685	1902	-0.1743 [-0.6148 - 0.2367]
SPTA1	1865	2103	-0.1734 [-0.4385 - 0.0744]
GRM1	2879	3237	-0.1689 [-0.4672 - 0.1222]
CA10	1268	1425	-0.1688 [-0.7475 - 0.4214]
PLPPR4	2635	2942	-0.1594 [-0.5217 - 0.2146]
USP29	2554	2841	-0.1532 [-0.3335 - 0.0195]
PCDH15	2946	3269	-0.1501 [-0.4269 - 0.0754]
CACNA1E	2373	2622	-0.1437 [-0.6275 - 0.3014]
DDI1	3026	3338	-0.1415 [-0.4045 - 0.0819]
RET	2835	3119	-0.1379 [-0.3289 - 0.0316]
SLC18A3	2837	3115	-0.1351 [-0.4001 - 0.1083]
FAM135B	3098	3388	-0.1291 [-0.2398 - -0.0273]

MAP2	1851	2023	-0.1284 [-0.8752 - 0.7147]
EGFLAM	1498	1633	-0.1242 [-0.7174 - 0.5127]
HS3ST4	2468	2680	-0.1192 [-0.3 - 0.0486]
ERBB2	2743	2971	-0.1153 [-0.3626 - 0.0904]
GBA3	1830	1981	-0.1147 [-0.4588 - 0.2468]
NEUROD4	2519	2722	-0.1119 [-0.4326 - 0.2172]
HCRTR2	1284	1386	-0.1111 [-1.0325 - 1.0075]
FBN2	1582	1704	-0.1074 [-0.3224 - 0.0941]
GRM8	2724	2935	-0.1072 [-0.4281 - 0.2251]
HECW1	2702	2905	-0.1042 [-0.427 - 0.1607]
USH2A	1902	2042	-0.1026 [-0.2881 - 0.0817]
CSMD3	1374	1475	-0.1024 [-0.4852 - 0.2299]
SOX9	2621	2800	-0.0951 [-0.3378 - 0.1039]
SLITRK1	4205	4481	-0.0919 [-0.3037 - 0.097]
LRRC7	2859	3035	-0.0863 [-0.3402 - 0.1673]
C6orf118	2681	2840	-0.0834 [-0.4953 - 0.3181]
RALYL	1204	1274	-0.0814 [-0.4376 - 0.2676]
FOXG1	3602	3805	-0.0792 [-0.4243 - 0.2799]
KCNC2	1883	1985	-0.0765 [-0.5759 - 0.4735]
ZIC1	1566	1650	-0.0755 [-0.3332 - 0.1848]
KCTD8	1657	1743	-0.0732 [-0.4623 - 0.3196]
TNR	2033	2138	-0.0731 [-0.5369 - 0.3928]
KIT	1930	2029	-0.0718 [-0.2374 - 0.098]
ASTN1	1665	1733	-0.0574 [-0.3976 - 0.218]
HS3ST5	2369	2465	-0.0573 [-0.1506 - 0.0297]
CHRM2	2869	2982	-0.0555 [-0.2062 - 0.0941]
ITSN1	2001	2079	-0.0553 [-0.2648 - 0.1422]
FAT1	1906	1975	-0.0512 [-0.6886 - 0.6484]
LRRTM1	2978	3081	-0.0491 [-0.3393 - 0.2162]
NFE2L2	1364	1410	-0.0472 [-0.4555 - 0.3443]
PAX6	1735	1792	-0.0463 [-0.4118 - 0.3105]
ZNF521	3481	3595	-0.0463 [-0.3145 - 0.1815]
FBXL7	2886	2972	-0.0419 [-0.3015 - 0.1953]
GRM5	2329	2397	-0.0414 [-0.2597 - 0.1488]
KRAS	1559	1604	-0.0409 [-0.3385 - 0.2337]
PDGFRA	1768	1814	-0.0366 [-0.1168 - 0.0404]
POLE	2765	2834	-0.0354 [-0.3765 - 0.2531]
ROS1	2583	2641	-0.0321 [-0.2543 - 0.1546]
ST6GALNAC3	1288	1315	-0.0302 [-0.2417 - 0.1651]
NYAP2	2690	2745	-0.0293 [-0.3082 - 0.2354]
NMUR1	1943	1971	-0.0205 [-0.2473 - 0.1951]
HCN1	3685	3716	-0.0123 [-0.3173 - 0.2497]
DMD	1202	1213	-0.0122 [-0.4888 - 0.3953]
CDKN2A	1995	2010	-0.0105 [-0.3876 - 0.3242]
NAV3	2766	2776	-0.0049 [-0.2263 - 0.2087]
CDH18	1614	1619	-0.0044 [-0.5172 - 0.4937]
POM121L12	2467	2470	-0.0016 [-0.3264 - 0.2714]
KCNJ3	1852	1850	0.0015 [-0.1791 - 0.1798]
TMEM200A	2959	2938	0.0104 [-0.2559 - 0.2585]
GRIA2	1549	1537	0.0117 [-0.5753 - 0.6706]

TRIM58	3117	3085	0.015 [-0.2615 - 0.2775]
HEBP1	1748	1727	0.0172 [-0.4778 - 0.529]
ADAMTS12	1364	1347	0.0181 [-0.5044 - 0.5892]
BRINP2	2934	2895	0.0194 [-0.3209 - 0.2785]
MET	1829	1803	0.0207 [-0.0879 - 0.1284]
FCRL5	1486	1465	0.021 [-0.3245 - 0.3048]
BRCA1	2004	1973	0.0223 [-0.1334 - 0.1702]
IL7R	2126	2086	0.0278 [-0.1725 - 0.2236]
BRCA2	1949	1901	0.0361 [-0.1326 - 0.1893]
NLRP3	3812	3679	0.0514 [-0.2037 - 0.2964]
DSC3	1229	1181	0.0566 [-0.6205 - 0.756]
CTNND2	1856	1767	0.0711 [-0.1229 - 0.2562]
LRFN5	2295	2184	0.0715 [-0.155 - 0.2899]
PREX1	2679	2538	0.0784 [-0.3741 - 0.5585]
DPYD	1538	1437	0.0986 [-0.1316 - 0.3068]
DOCK3	1703	1589	0.0997 [-0.3671 - 0.4974]
BRAF	1780	1654	0.1058 [-0.1103 - 0.2934]
TIAM1	3503	3255	0.106 [-0.0898 - 0.2764]
GJA8	3245	3006	0.1107 [-0.1632 - 0.3511]
NXPH4	1691	1566	0.111 [-0.4037 - 0.6735]
DCSTAMP	3153	2910	0.1156 [-0.1925 - 0.4043]
CDH9	1770	1632	0.1177 [-0.067 - 0.279]
FBXW7	2251	2073	0.1193 [-0.4836 - 0.7276]
PHACTR1	2333	2134	0.1283 [-0.462 - 0.7006]
GALNT17	1798	1645	0.1285 [-0.3861 - 0.6921]
CRACD	4854	4412	0.1377 [-0.0768 - 0.323]
ARFGEF1	1574	1424	0.1442 [-0.3836 - 0.6756]
CDH12	1672	1513	0.1444 [-0.1209 - 0.3883]
CPXCR1	1898	1713	0.1477 [-0.5358 - 0.8611]
PGK2	3237	2893	0.1621 [-0.0401 - 0.3379]
HTR1A	3303	2945	0.1655 [-0.1232 - 0.4384]
CDH8	1261	1121	0.1699 [-0.1631 - 0.4523]
ITGA10	2269	2015	0.1711 [-0.3173 - 0.6222]
SMAD4	1797	1596	0.1715 [-0.4199 - 0.7885]
THSD7A	2665	2364	0.1728 [-0.1596 - 0.4776]
NRAS	2112	1864	0.1808 [-0.0593 - 0.4055]
TNFRSF21	2681	2361	0.1831 [-0.3369 - 0.7404]
PKHD1L1	2827	2483	0.1874 [-0.0585 - 0.426]
APC	2616	2277	0.1997 [-0.048 - 0.4079]
SLPI	1353	1172	0.2073 [-0.0344 - 0.4364]
DCAF12L1	3082	2615	0.2371 [-0.3168 - 0.7902]
PIK3CA	1633	1381	0.2418 [-0.0423 - 0.5062]
EGFR	1237	1043	0.247 [-0.145 - 0.5327]
P2RY10	2670	2239	0.2538 [-0.3124 - 0.842]
TP53	3950	3258	0.2779 [-0.152 - 0.6169]
WIPF1	4112	3344	0.2981 [-0.2845 - 0.9085]
KEAP1	2972	2401	0.3077 [0.0177 - 0.5684]
RIN3	2430	1937	0.3276 [-0.0678 - 0.6985]
DCAF12L2	3239	2581	0.3277 [-0.2301 - 0.8987]
ZC3H12A	2512	2001	0.3282 [-0.1913 - 0.8708]

STK11	1988	1564	0.3462 [-0.023 - 0.7289]
BRINP3	3220	2514	0.3572 [0.0515 - 0.6215]
SLITRK4	2700	2092	0.3679 [-0.1807 - 0.8993]
CNTN5	1483	1147	0.371 [0.0416 - 0.6393]
CTNNB1	2136	1631	0.389 [-0.017 - 0.7065]
ZFPM2	4587	3455	0.4089 [0.0547 - 0.6784]
HTR2C	1759	1324	0.41 [-0.183 - 0.8608]
LRP1B	1659	1231	0.4305 [0.045 - 0.7786]
GBP7	1485	1096	0.4373 [0.0519 - 0.8182]
FRYL	1497	1097	0.4483 [-0.0118 - 0.9286]
ABCC5	2339	1690	0.4687 [0.0153 - 0.8969]
SV2A	2274	1615	0.4941 [-0.2397 - 1.3295]
VPS13B	2393	1677	0.5129 [0.0298 - 0.9941]
KLHL31	1665	1143	0.5428 [0.0044 - 0.9067]
CYBB	1710	1034	0.726 [-0.1634 - 1.6955]
MAP7D3	2207	1318	0.7439 [-0.0898 - 1.5554]
