# Supplementary material for: Cell‐free chromatin immunoprecipitation can determine tumor gene expression in lung cancer patients
Source: Mol Oncol. 2023 Mar 5;17(5):722–36. doi: 10.1002/1878-0261.13394 (PMC10158780; doi:10.1002/1878-0261.13394)
Supplement: Supplementary file 9 — Table S8. Average enrichment in EGFR‐mut (n = 2) and EGFR‐WT (n = 6) NSCLC patients. [file MOL2-17-722-s008.pdf]

Table. S8. Average enrichment in EGFR-mut (n = 2) and EGFR-WT (n = 6) NSCLC patients.
For each gene the Log2FC between EGFR-mut and EGFR-WT patients and 95% confidence interval is calculated

SYMBOL	Average EGFR-mut enrichment	Average EGFR-WT enrichment	Log2FC [95% CI]
KLHL31	1357	1939	-0.5151 [-1.1835 - 0.4023]
SMAD4	1484	2103	-0.5032 [-1.921 - 0.9739]
CTNNB1	1840	2588	-0.4916 [-1.782 - 0.9306]
FRYL	1298	1815	-0.4831 [-0.7913 - -0.1199]
DSC3	973	1336	-0.4575 [-0.8979 - 0.1241]
CSMD1	860	1156	-0.4269 [-2.8336 - 2.3092]
FBXW7	1948	2562	-0.3956 [-1.4114 - 0.7535]
KRAS	1288	1693	-0.3939 [-1.9539 - 1.1694]
TP53	3561	4674	-0.3924 [-1.2389 - 0.66]
ZNF521	2835	3696	-0.3827 [-6.2627 - 5.3509]
CDH8	1065	1379	-0.3736 [-1.8927 - 1.1859]
TRIM58	2606	3372	-0.3719 [-2.2485 - 1.4946]
CNTN5	1341	1714	-0.3534 [-4.3134 - 3.5393]
RET	2269	2894	-0.3511 [-1.3306 - 0.6342]
SLITRK1	3477	4386	-0.335 [-3.5902 - 2.8709]
DDI1	2502	3148	-0.3312 [-5.1829 - 4.4148]
GRM5	1967	2456	-0.3203 [-2.0541 - 1.4126]
GBP7	1397	1735	-0.3127 [-5.4836 - 4.7391]
HTR1A	2929	3637	-0.3126 [-5.3307 - 4.5959]
CRACD	4369	5410	-0.3083 [-1.8145 - 1.2127]
KCNA5	1754	2165	-0.3036 [-2.3849 - 1.8801]
APC	2404	2956	-0.298 [-1.5973 - 1.0446]
NRAS	1919	2347	-0.29 [-0.8024 - 0.2635]
CYBB	1749	2113	-0.2727 [-2.365 - 1.9872]
PREX1	2412	2897	-0.2643 [-1.5809 - 1.0553]
DPYD	1395	1675	-0.2642 [-0.9287 - 0.4346]
PAX6	1498	1796	-0.2611 [-4.8169 - 4.2007]
ST6GALNAC3	1138	1349	-0.2453 [-3.1995 - 2.6688]
HS3ST4	2102	2489	-0.2436 [-2.2407 - 1.7356]
BRCA2	1760	2077	-0.239 [-1.8982 - 1.4101]
PIK3CA	1574	1835	-0.2213 [-0.6167 - 0.278]
NXPH4	1535	1788	-0.2199 [-0.5477 - 0.1255]
HEBP1	1571	1829	-0.2192 [-0.7999 - 0.4487]
LRP1B	1650	1914	-0.2143 [-2.9651 - 2.5395]
MYH7	1465	1697	-0.2126 [-0.7074 - 0.3182]
NLRP3	3540	4081	-0.2052 [-0.7389 - 0.3705]
WSCD2	1093	1248	-0.1911 [-1.3518 - 1.8884]
PCDH15	2534	2885	-0.1875 [-0.7319 - 0.5137]
TMEM200A	2718	3095	-0.1873 [-5.5299 - 5.0317]
C6orf118	2397	2728	-0.1865 [-3.6125 - 3.1906]
NEUROD4	2243	2540	-0.1793 [-1.0022 - 0.6552]
ABCC5	2369	2673	-0.1741 [-0.6502 - 0.4329]

ZC3H12A	2507	2828	-0.1734 [-0.5884 - 0.3618]
NAV3	2500	2818	-0.173 [-0.7826 - 0.4715]
FAT1	1674	1886	-0.1716 [-0.6659 - 0.4759]
ALK	1817	2043	-0.1693 [-0.5665 - 0.2962]
PDZRN3	2402	2697	-0.1676 [-2.4675 - 2.1149]
LRRTM1	2693	2983	-0.1473 [-0.8548 - 0.6264]
RIN3	2495	2747	-0.1387 [-0.6061 - 0.468]
NFE2L2	1257	1382	-0.1372 [-0.5443 - 0.3852]
LRRC7	2607	2849	-0.1282 [-3.9206 - 3.6016]
SLC39A12	1486	1622	-0.1271 [-1.8058 - 1.8697]
POLE	2571	2797	-0.1217 [-0.9188 - 0.834]
ZIC4	2714	2952	-0.1212 [-2.1055 - 1.8446]
GRIN2B	1475	1600	-0.1172 [-3.7777 - 3.491]
BRAF	1759	1907	-0.1161 [-0.4639 - 0.3052]
HTR1E	2267	2450	-0.1119 [-0.6847 - 0.548]
GBA3	1697	1831	-0.1094 [-2.6664 - 2.4172]
ROS1	2396	2576	-0.1045 [-0.4907 - 0.3733]
ZIC1	1457	1565	-0.1034 [-3.4773 - 3.2201]
FBN2	1458	1566	-0.103 [-1.2979 - 1.1066]
KCNJ3	1780	1907	-0.0994 [-0.3114 - 0.1334]
GRM8	2477	2648	-0.0965 [-1.0121 - 0.8355]
KEAP1	3079	3281	-0.0914 [-0.5847 - 0.4973]
PDYN	2185	2320	-0.0868 [-0.5442 - 0.5078]
GRIN3B	1954	2066	-0.0804 [-0.4707 - 0.4049]
DOCK3	1634	1724	-0.0771 [-1.7005 - 1.6784]
CHRM2	2732	2871	-0.0719 [-0.4776 - 0.3428]
PGK2	3275	3440	-0.0707 [-0.9392 - 0.8385]
FAM71B	2324	2440	-0.0703 [-0.6992 - 0.6157]
PDGFRA	1692	1770	-0.0646 [-1.4547 - 1.3164]
USP29	2366	2463	-0.0582 [-2.4121 - 2.2709]
SV2A	2441	2533	-0.0533 [-2.6256 - 2.4971]
POM121L12	2423	2496	-0.043 [-1.1718 - 1.1824]
NMUR1	1899	1956	-0.0421 [-0.7496 - 0.6922]
GRM1	2658	2732	-0.0394 [-0.8256 - 0.7993]
GPR139	2137	2196	-0.0388 [-0.7846 - 0.7887]
KCNC2	1825	1871	-0.0362 [-0.5526 - 0.4846]
GRIA2	1542	1571	-0.0268 [-0.2745 - 0.2499]
CRMP1	1141	1158	-0.0218 [-0.9252 - 0.9519]
WIPF1	4431	4499	-0.0218 [-0.4811 - 0.5781]
KCTD8	1586	1604	-0.0165 [-0.8952 - 0.923]
USH2A	1858	1867	-0.0071 [-0.2394 - 0.2474]
ARFGEF1	1642	1644	-0.0023 [-0.7973 - 0.8723]
FOXG1	3577	3572	0.002 [-0.6926 - 0.7126]
RALYL	1183	1178	0.0065 [-1.2041 - 1.2556]
ACTN2	1024	1012	0.0166 [-0.5796 - 0.8324]
DMD	1200	1182	0.0206 [-3.0449 - 3.1195]
ADAMTS12	1357	1333	0.026 [-0.3868 - 0.4761]

VPS13B	2695	2646	0.0261 [-3.9151 - 3.8982]
PLPPR4	2541	2489	0.0298 [-0.3472 - 0.4815]
HS3ST5	2349	2290	0.0367 [-0.3283 - 0.4239]
SLC18A3	2803	2729	0.0389 [-0.4084 - 0.55]
STK11	2190	2129	0.0406 [-1.7686 - 1.8427]
TBXT	1832	1730	0.083 [-0.4733 - 0.8372]
RNASE3	1003	945	0.0858 [-0.4172 - 0.7078]
FAM135B	3091	2902	0.0912 [-0.163 - 0.378]
CTNND2	1994	1869	0.0935 [-1.4459 - 1.6315]
GRIK3	1743	1630	0.0962 [-0.6022 - 1.0715]
MET	1936	1804	0.1015 [-0.6452 - 0.8464]
BRCA1	2120	1973	0.1039 [-0.5322 - 0.7505]
UGT3A2	1636	1521	0.1053 [-0.5182 - 0.9898]
GALNT17	1937	1799	0.1067 [-0.356 - 0.5853]
IL7R	2251	2089	0.1077 [-0.9306 - 1.1497]
KIT	2012	1858	0.1148 [0.0441 - 0.188]
MAP2	1885	1738	0.117 [-1.4478 - 1.7514]
CPXCR1	2102	1932	0.1218 [-1.435 - 1.8077]
SLPI	1493	1371	0.123 [-0.1713 - 0.4725]
EGFLAM	1513	1388	0.1243 [-1.2555 - 1.5448]
CDH9	1945	1759	0.1452 [-0.5668 - 0.885]
ADAMTS16	1688	1515	0.1557 [-0.0862 - 0.4314]
KIF19	1384	1240	0.1581 [-0.8574 - 1.6438]
FAM151A	1284	1147	0.1632 [-0.4194 - 0.9694]
MYT1L	1322	1180	0.1635 [-0.7902 - 1.6644]
CNTNAP2	1308	1166	0.165 [-0.2647 - 0.7127]
FCRL5	1662	1478	0.1692 [-1.2854 - 1.7279]
BRINP3	3724	3294	0.1772 [-2.9603 - 3.2806]
C6	1246	1102	0.1773 [-0.5212 - 1.1589]
NYAP2	2861	2525	0.1803 [-0.2098 - 0.6617]
MMP16	1272	1122	0.1809 [-0.4829 - 0.9767]
TNFRSF21	3136	2744	0.1928 [-1.9378 - 2.3122]
SPTA1	1990	1729	0.2023 [-0.3605 - 0.8469]
LRFN5	2574	2233	0.2049 [-0.1196 - 0.5653]
PKHD1L1	3254	2818	0.2076 [-3.2411 - 3.6039]
HCN1	3981	3441	0.2101 [-0.7323 - 1.2799]
TNR	2144	1846	0.2161 [-0.6268 - 1.1917]
ROBO2	1015	862	0.2359 [-0.9325 - 2.2339]
ZFPM2	5746	4872	0.2378 [-1.2547 - 1.8223]
DSCAM	1770	1493	0.2457 [-0.593 - 1.4326]
ABCG2	969	814	0.2525 [-1.2574 - 1.8776]
MAP7D3	2919	2428	0.2659 [-0.5678 - 1.4747]
TIAM1	4108	3405	0.2709 [-2.5685 - 3.0729]
ITSN1	2262	1862	0.281 [-1.0929 - 1.6522]
FBXL7	3273	2654	0.3025 [-4.3603 - 4.8686]
KPRP	3478	2789	0.3185 [-1.6172 - 2.3555]
SEMA5B	1703	1364	0.3197 [-0.7994 - 1.7705]

CDH12	1995	1597	0.3208 [-0.1904 - 0.867]
P2RY10	3320	2636	0.3327 [-0.1078 - 0.8963]
PHACTR1	2918	2277	0.358 [-0.3423 - 1.2344]
CDH18	1857	1447	0.36 [-0.5285 - 1.4092]
ERBB2	3137	2442	0.3613 [-4.5498 - 5.163]
HCRTR2	1507	1166	0.3701 [-0.4023 - 1.1559]
SOX9	3012	2324	0.3742 [-4.6769 - 5.3068]
DCAF12L2	4160	3195	0.3808 [-0.1133 - 1.0009]
CPZ	1620	1227	0.4016 [-0.2355 - 1.3673]
DCSTAMP	3940	2956	0.4146 [-3.3617 - 4.1273]
HECW1	3178	2325	0.451 [-1.4581 - 2.4153]
GJA8	4101	2979	0.4611 [-3.1957 - 4.057]
THSD7A	3445	2483	0.4724 [-2.9616 - 3.8518]
ITGA10	2912	2099	0.4725 [-1.8596 - 2.8286]
SLITRK4	3685	2621	0.4918 [-0.0714 - 1.1807]
CSMD3	1638	1163	0.4937 [-0.4943 - 1.7494]
CA10	1478	1043	0.5031 [-0.525 - 2.0379]
DCAF12L1	4079	2876	0.5041 [0.0206 - 1.1331]
CACNA1E	2863	1999	0.5182 [-2.6079 - 3.6543]
CDKN2A	2551	1778	0.5205 [-1.9486 - 2.9787]
ASTN1	2085	1434	0.5403 [-1.5524 - 2.6517]
BRINP2	3777	2532	0.5769 [-3.618 - 4.7011]
HTR2C	2679	1598	0.7449 [-0.1684 - 1.9315]
EGFR	1889	1068	0.8225 [-3.7379 - 5.2903]
