# Supplementary material for: Cell‐free chromatin immunoprecipitation can determine tumor gene expression in lung cancer patients
Source: Mol Oncol. 2023 Mar 5;17(5):722–36. doi: 10.1002/1878-0261.13394 (PMC10158780; doi:10.1002/1878-0261.13394)
Supplement: Supplementary file 10 — Table S9. Average enrichment in NSCLC (n = 8) and SCLC (n = 4) patients. [file MOL2-17-722-s006.pdf]

Table. S9. Average enrichment in NSCLC (n = 8) and SCLC (n = 4) patients.

For each gene the Log2FC between NSCLC and SCLC patients and 95% confidence interval is calculated

SYMBOL	Average NSCLC enrichemt	Average SCLC enrichment	Log2FC [95% CI]
KIF19	1276	2563	-1.006 [-2.1008 - -0.1442]
CRMP1	1154	1860	-0.689 [-1.3716 - 0.0319]
PAX6	1721	2753	-0.6774 [-1.4243 - 0.1766]
MYT1L	1215	1944	-0.6773 [-1.7484 - 0.042]
GRIK3	1658	2453	-0.5647 [-1.3721 - 0.1297]
SMAD4	1948	2823	-0.5351 [-1.3829 - 0.3652]
SOX9	2496	3459	-0.4711 [-1.3793 - 0.5811]
RALYL	1179	1620	-0.4581 [-1.0586 - 0.1683]
PDZRN3	2624	3576	-0.4469 [-1.1471 - 0.3492]
CACNA1E	2215	3005	-0.4397 [-1.1388 - 0.1739]
MAP2	1775	2392	-0.4304 [-1.066 - 0.1651]
SEMA5B	1449	1931	-0.414 [-1.3256 - 0.2327]
PLPPR4	2502	3327	-0.4114 [-0.7175 - -0.1601]
GRIN3B	2038	2710	-0.4109 [-1.0424 - 0.2469]
ACTN2	1015	1344	-0.405 [-0.9853 - 0.0301]
LRRC7	2789	3670	-0.3963 [-0.7368 - -0.0482]
WSCD2	1210	1558	-0.3656 [-1.8016 - 0.4333]
CSMD1	1082	1387	-0.3589 [-1.4724 - 0.3277]
NXPH4	1725	2197	-0.3493 [-1.0882 - 0.5207]
BRCA1	2010	2559	-0.3486 [-0.6658 - -0.0253]
HCN1	3576	4549	-0.3473 [-1.1535 - 0.4819]
PHACTR1	2437	3068	-0.3324 [-0.9287 - 0.1304]
KEAP1	3230	4025	-0.3172 [-1.0535 - 0.4911]
CA10	1152	1428	-0.3102 [-1.4177 - 0.3905]
ZIC4	2892	3580	-0.3079 [-0.6339 - 0.0312]
EGFLAM	1419	1753	-0.3052 [-0.7074 - 0.0482]
ASTN1	1597	1971	-0.3042 [-0.832 - 0.1447]
KCNA5	2062	2538	-0.2999 [-0.9099 - 0.1475]
ABCG2	853	1035	-0.2797 [-0.9006 - 0.216]
ABCC5	2597	3150	-0.2789 [-0.9465 - 0.3916]
BRINP2	2844	3447	-0.2774 [-0.8783 - 0.2493]
ITSN1	1962	2376	-0.2762 [-0.6273 - 0.0727]
PIK3CA	1769	2135	-0.2707 [-0.7587 - 0.1801]
GRM8	2606	3139	-0.2689 [-0.6888 - 0.1615]
ZNF521	3481	4192	-0.2682 [-0.6395 - 0.0625]
POLE	2741	3300	-0.2679 [-0.8664 - 0.2371]
GPR139	2181	2618	-0.2633 [-0.6193 - 0.0111]
CDKN2A	1972	2355	-0.2566 [-0.8032 - 0.2511]
NYAP2	2609	3059	-0.2296 [-0.603 - 0.0844]
KIT	1897	2221	-0.228 [-0.5064 - 0.0649]
SV2A	2510	2936	-0.2265 [-0.8727 - 0.475]
MMP16	1159	1348	-0.2177 [-0.7112 - 0.1733]
CNTNAP2	1202	1394	-0.2145 [-0.6111 - 0.0977]
TNR	1921	2217	-0.2072 [-0.8114 - 0.3279]
UGT3A2	1549	1788	-0.2067 [-0.8439 - 0.2593]

RNASE3	960	1107	-0.2057 [-0.6799 - 0.2062]
ZIC1	1538	1770	-0.203 [-0.4319 - 0.0173]
LRFN5	2318	2656	-0.1966 [-0.7856 - 0.4438]
CPZ	1325	1512	-0.1905 [-1.0416 - 0.5142]
GRIN2B	1568	1780	-0.1826 [-0.5591 - 0.1325]
SLC39A12	1588	1793	-0.1753 [-1.0975 - 0.45]
FAM151A	1181	1333	-0.1748 [-0.7739 - 0.281]
STK11	2144	2414	-0.1706 [-0.6784 - 0.3706]
PDYN	2287	2563	-0.1649 [-0.6615 - 0.2692]
FCRL5	1524	1700	-0.1575 [-0.8638 - 0.5175]
KPRP	2962	3297	-0.1547 [-0.9524 - 0.6084]
FBXL7	2809	3107	-0.1456 [-0.4044 - 0.0638]
CRACD	5150	5659	-0.1359 [-0.5184 - 0.2095]
ADAMTS16	1559	1709	-0.1327 [-0.3437 - 0.053]
NFE2L2	1351	1480	-0.1315 [-0.5882 - 0.268]
NEUROD4	2466	2694	-0.1281 [-0.528 - 0.2833]
FAM135B	2949	3200	-0.1177 [-0.7165 - 0.5361]
DMD	1187	1284	-0.114 [-0.7521 - 0.4127]
TBXT	1755	1895	-0.1103 [-0.6639 - 0.3164]
CDH8	1300	1402	-0.1088 [-0.6674 - 0.4166]
HEBP1	1764	1892	-0.1006 [-0.5514 - 0.2957]
FAM71B	2411	2583	-0.0999 [-0.6935 - 0.5195]
ADAMTS12	1339	1431	-0.0956 [-0.3652 - 0.151]
FOXG1	3573	3812	-0.0933 [-1.1629 - 1.3009]
ROBO2	901	954	-0.0838 [-1.4955 - 0.7074]
KRAS	1591	1683	-0.0805 [-0.3939 - 0.1802]
HTR1E	2405	2538	-0.0781 [-0.4495 - 0.2246]
ST6GALNAC3	1296	1367	-0.0764 [-0.4057 - 0.2388]
NAV3	2738	2885	-0.075 [-0.5561 - 0.4179]
GRM5	2333	2453	-0.0722 [-0.4561 - 0.2874]
C6	1138	1195	-0.0702 [-0.7726 - 0.4334]
CDH9	1805	1884	-0.0615 [-0.3957 - 0.2549]
KCNC2	1860	1941	-0.0613 [-0.7237 - 0.6952]
CSMD3	1282	1337	-0.0601 [-0.929 - 0.6233]
HS3ST5	2305	2401	-0.0592 [-0.3467 - 0.2256]
NMUR1	1942	2022	-0.0588 [-0.49 - 0.3798]
TIAM1	3581	3724	-0.0566 [-0.5794 - 0.5027]
C6orf118	2646	2724	-0.042 [-0.3888 - 0.2351]
DSCAM	1562	1604	-0.0374 [-0.8811 - 0.5678]
BRAF	1870	1907	-0.0283 [-0.5764 - 0.5307]
ALK	1986	2013	-0.0192 [-0.6566 - 0.6571]
CDH18	1549	1569	-0.018 [-0.5729 - 0.3674]
USP29	2439	2466	-0.0157 [-0.2889 - 0.2523]
SLITRK4	2887	2918	-0.0154 [-0.6766 - 0.5855]
DSC3	1245	1259	-0.0152 [-0.6284 - 0.539]
RET	2738	2765	-0.0146 [-0.2799 - 0.2243]
IL7R	2129	2148	-0.0125 [-0.3101 - 0.2851]
CTNND2	1900	1913	-0.0094 [-0.3516 - 0.3284]
USH2A	1865	1863	0.0011 [-0.21 - 0.2026]
DDI1	2986	2978	0.0039 [-0.431 - 0.4129]

NRAS	2240	2223	0.011 [-0.4696 - 0.4933]
KCTD8	1600	1584	0.0141 [-0.6386 - 0.693]
GBA3	1797	1777	0.0163 [-0.3013 - 0.3383]
SLPI	1402	1379	0.0239 [-0.3033 - 0.3276]
HECW1	2538	2491	0.027 [-0.5388 - 0.4921]
SLC18A3	2747	2689	0.0311 [-0.2729 - 0.2866]
GRM1	2713	2650	0.034 [-0.2973 - 0.3151]
CDH12	1697	1653	0.0381 [-0.3093 - 0.3575]
HS3ST4	2392	2328	0.0392 [-0.3157 - 0.3995]
MET	1837	1787	0.0393 [-0.2067 - 0.294]
CPXCR1	1974	1917	0.0428 [-0.5338 - 0.4604]
PCDH15	2797	2708	0.0468 [-0.4495 - 0.4362]
LRRTM1	2910	2810	0.0509 [-0.3259 - 0.3751]
FRYL	1685	1627	0.0512 [-0.3767 - 0.4421]
HTR2C	1868	1785	0.0659 [-0.8522 - 0.7519]
KCNJ3	1875	1791	0.0662 [-0.4084 - 0.5752]
GJA8	3260	3097	0.0739 [-0.3223 - 0.4455]
FAT1	1833	1741	0.0746 [-0.4484 - 0.5201]
ZC3H12A	2748	2596	0.082 [-0.7009 - 0.9372]
MYH7	1639	1544	0.0859 [-0.1642 - 0.2968]
ARFGEF1	1643	1540	0.0939 [-0.485 - 0.6564]
DCAF12L1	3177	2968	0.0984 [-0.5594 - 0.6871]
SPTA1	1794	1670	0.1036 [-0.4122 - 0.5953]
DCAF12L2	3436	3197	0.104 [-0.4046 - 0.5227]
PDGFRA	1751	1628	0.1047 [-0.3077 - 0.5487]
PKHD1L1	2927	2715	0.1086 [-0.4495 - 0.7188]
POM121L12	2478	2295	0.1101 [-0.4582 - 0.6194]
ERBB2	2616	2419	0.113 [-0.2956 - 0.4726]
CHRM2	2837	2579	0.1371 [-0.1614 - 0.4445]
BRINP3	3401	3088	0.1392 [-0.3522 - 0.6168]
PREX1	2776	2520	0.1393 [-0.2228 - 0.4922]
TNFRSF21	2842	2579	0.1402 [-0.2394 - 0.5044]
FBN2	1539	1395	0.1412 [-0.3384 - 0.6328]
APC	2818	2551	0.1435 [-0.3294 - 0.5734]
NLRP3	3946	3570	0.1444 [-0.2683 - 0.5494]
DPYD	1605	1445	0.1519 [-0.236 - 0.5199]
BRCA2	1998	1792	0.1572 [-0.1788 - 0.4897]
GALNT17	1834	1633	0.1671 [-0.0702 - 0.3954]
HCRTR2	1251	1111	0.1715 [-0.1125 - 0.425]
GRIA2	1564	1385	0.175 [-0.1213 - 0.4675]
TMEM200A	3001	2646	0.1817 [-0.181 - 0.5245]
FBXW7	2409	2114	0.188 [-0.4226 - 0.6943]
TP53	4395	3844	0.1933 [-0.5808 - 0.8731]
CNTN5	1621	1416	0.1945 [-0.3906 - 0.7591]
DCSTAMP	3202	2794	0.1966 [-0.2041 - 0.5822]
GBP7	1651	1438	0.199 [-0.1417 - 0.4813]
ROS1	2531	2204	0.1998 [-0.1819 - 0.5302]
WIPF1	4482	3902	0.2001 [-0.2972 - 0.6297]
P2RY10	2807	2427	0.2101 [-0.493 - 0.9205]
CYBB	2022	1742	0.2149 [-0.5501 - 0.7914]

CTNNB1	2401	2023	0.2471 [-0.6113 - 1.072]
TRIM58	3180	2671	0.2517 [-0.2635 - 0.7902]
ITGA10	2302	1932	0.2526 [-0.2553 - 0.6315]
THSD7A	2723	2283	0.2541 [-0.051 - 0.5039]
PGK2	3399	2846	0.2558 [-0.2155 - 0.7266]
DOCK3	1702	1404	0.2771 [-0.3875 - 0.8084]
HTR1A	3460	2845	0.2825 [-0.2414 - 0.8209]
SLITRK1	4159	3348	0.3127 [-0.3238 - 1.0034]
KLHL31	1793	1364	0.3949 [-0.3647 - 1.005]
LRP1B	1848	1369	0.4326 [-0.1075 - 0.9036]
EGFR	1273	937	0.4431 [-0.0011 - 0.7601]
VPS13B	2658	1876	0.5027 [0.0649 - 0.8949]
MAP7D3	2550	1746	0.5463 [-0.3274 - 1.175]
ZFPM2	5091	3367	0.5962 [-0.0299 - 1.156]
RIN3	2684	1754	0.6133 [-0.246 - 1.5717]
