# Supplementary material for: Cell‐free chromatin immunoprecipitation can determine tumor gene expression in lung cancer patients
Source: Mol Oncol. 2023 Mar 5;17(5):722–36. doi: 10.1002/1878-0261.13394 (PMC10158780; doi:10.1002/1878-0261.13394)
Supplement: Supplementary file 11 — Table S10. Raw unique read counts for all ChIP and cfChIP samples. [file MOL2-17-722-s001.pdf]

Table S10. Raw unique read counts for all cfChIP samples.

Genes	Raw unique readcounts																							
	NAC.1	NAC.2	NAC.3	NAC.4	NSC.1	NSC.2	NSC.3	NSC.4	SCC.1	SCC.2	SCC.3	SCC.4	HC.1	HC.2	HC.3	HC.4	A549.R1	A549.R2	A549.R3	HCC827.R1	HCC827.R2	HCC827.R3	HCC827-MET.R1	HCC827-MET.R2	HCC827-MET.R3
ABCC5	1872	1367	502	1224	1195	431	1331	681	9776	1857	12969	2742	132	108	243	144	5574	4177	4802	4462	4502	5005	3299	3858	3218
ABCG2	583	817	147	644	374	170	530	258	3580	881	2060	1131	102	108	89	116	6985	6010	6162	779	921	966	758	1056	678
ACTN2	777	1242	262	512	327	150	511	245	3930	1218	3483	989	83	158	96	95	849	686	605	364	346	443	288	440	274
ADAMTS12	1053	1152	267	697	362	216	820	512	4458	1314	3679	1276	69	97	176	134	3165	2748	2854	329	354	319	229	434	275
ADAMTS16	1204	1298	371	910	419	270	914	519	5295	1325	4705	1559	94	148	272	136	3628	2961	3277	4896	4976	5305	3862	4632	3906
ALK	30072	51564	10871	25225	12498	7944	28588	16403	150880	50446	82770	45025	4241	5015	6001	4214	55207	40889	42541	27346	28108	29829	18675	27051	17744
APC	31579	36220	11513	31672	22906	11857	29209	16342	225368	34789	131360	54007	3414	3658	5294	4404	160231	135363	133291	54550	58895	59214	38515	51943	37614
ARFGEF1	1476	827	376	865	586	294	1159	637	5837	968	5395	1804	96	135	134	178	9269	8630	7829	11981	13382	12851	7954	10084	8140
ASTN1	3910	3834	693	1960	791	580	1733	1036	13824	4453	10218	3409	299	309	363	323	2485	1634	1777	769	742	1118	905	1463	717
ASTN2	1468	2536	499	1221	673	403	1360	762	8333	2153	12492	2476	186	248	347	289	1479	920	979	665	664	910	583	897	509
AVPR1A	1984	3702	936	2324	1305	812	2058	946	13169	2715	10532	3270	304	361	409	329	3283	2686	2863	7297	7625	7991	3544	5304	2802
BCHE	4918	5365	1485	3916	2040	1062	4082	2724	20670	8256	17867	7495	470	574	741	639	5925	4369	4098	3495	3567	3740	1891	4144	1968
BPIFB4	2846	3436	657	1380	574	369	1479	836	8282	3244	5477	2635	316	262	306	311	1351	856	1035	645	595	744	449	724	371
BRAF	15292	12227	4321	11094	7926	4299	10911	6994	80478	12642	73864	21428	1511	1457	2134	1644	76132	66932	64010	53539	58819	54887	29889	39235	30356
BRCA1	47466	41998	11707	30660	21382	9960	29193	17528	228739	58575	237485	58141	3977	4208	6704	4877	508320	433199	438193	229336	247693	252335	162001	203559	163125
BRCA2	37350	43888	13894	35636	21937	12075	37411	21726	228063	39018	165700	55598	4499	4625	6848	5592	283927	254209	232183	180931	213852	200214	103947	134478	105716
BRINP2	8087	6923	1372	3397	1688	959	3292	1935	24002	7680	25687	5529	509	522	708	603	4086	2782	3051	1219	1186	1577	1075	1784	900
BRINP3	7323	5925	1351	3432	1886	1042	4105	2967	20669	7184	15047	6571	407	505	649	422	5693	4141	4286	3189	3398	3464	2240	3905	2074
C6	918	875	307	782	386	177	818	530	3500	1159	3222	1288	90	112	202	174	1221	862	766	261	279	314	134	286	115
C6orf118	1982	3927	898	2364	935	662	2176	1325	13070	3291	9776	3880	319	366	335	366	1558	1285	1256	420	321	519	308	573	352
CA10	1229	1171	291	710	339	141	817	343	4249	1477	3636	1221	70	147	159	125	1394	982	1116	397	285	435	194	484	226
CACNA1E	3235	3187	616	1402	726	399	1471	739	10491	4250	8571	2821	265	303	264	256	1220	755	961	457	368	540	394	656	367
CDH12	1584	1279	347	1007	439	255	1162	545	5162	1538	4052	1735	121	139	158	120	1585	1214	1176	333	286	489	197	451	189
CDH18	1573	1247	325	887	341	224	1137	641	5532	1208	4574	1547	122	128	235	103	1955	1584	1297	698	740	671	276	614	355
CDH8	750	1421	326	782	349	208	990	565	4386	1105	5885	1434	104	91	131	117	1678	1263	1304	450	444	602	206	609	254
CDH9	5510	4706	1361	3396	1573	1017	3957	2272	18806	5656	20342	6336	450	458	637	486	8724	7397	6816	1723	1800	1990	1006	2324	1226
CDKN2A	2656	2028	577	1324	648	416	991	606	8177	1833	9976	2787	142	217	280	213	37	10	59	20513	22442	21814	14880	16122	14676
CHRM2	4854	6364	1527	4027	2180	1410	4166	2084	25382	5837	14835	5769	498	600	883	650	5839	4444	4379	1318	1396	1849	1488	2729	1454
CNTN5	1540	2039	641	1949	956	575	2395	1235	8068	2746	6577	3562	200	185	278	197	2122	1292	1175	801	911	1107	660	1340	501
CNTNAP2	3082	3487	1041	2261	893	649	2201	1301	14113	3284	12490	4346	334	412	529	358	5433	4191	3951	1306	1146	1646	646	1331	606
CPXCR1	2071	1927	452	2329	985	688	2497	1050	12801	2333	8194	3336	230	175	241	370	3346	2472	2169	373	321	416	155	370	177
CPZ	1349	2093	365	1008	359	243	862	497	5220	2000	3144	1469	205	181	181	193	554	355	422	395	408	409	395	680	365
CRACD	11878	16133	5085	11863	8225	4123	10839	5727	76146	17310	80725	21920	1407	1593	1960	1646	8179	5862	6593	4424	4396	4504	6300	7546	5341
CRMP1	789	1443	280	757	314	246	680	443	4432	1290	7376	1745	150	153	173	120	1056	775	799	614	490	621	428	708	322
CSMD1	442	1335	297	624	280	138	746	373	4251	1258	2476	1316	123	82	160	116	1491	1063	1079	255	294	348	191	297	192
CSMD3	8076	6244	1683	4393	1862	944	5319	2860	22673	7629	14696	9132	714	675	1011	602	9358	7049	6335	1492	1308	1847	836	1945	991
CTNNB1	1098	1377	390	1164	1095	478	961	661	9421	956	6279	1716	133	119	193	124	9852	8674	9046	8327	8831	8621	5617	6817	5537
CTNND2	1745	1505	411	1005	644	396	1055	537	8291	1691	5387	1549	123	158	221	158	1320	861	1083	308	360	423	290	462	270
CYBB	971	966	296	1224	811	463	1233	408	7915	1015	4996	1844	51	77	97	164	982	886	832	331	387	424	188	483	201
DCAF12L1	7640	5010	1091	5735	2898	1404	5309	2396	33774	4434	21586	7970	431	479	674	856	4977	3474	4038	2062	1870	2155	1475	2473	1441
DCAF12L2	7730	5274	1101	4985	2581	1601	4964	2190	31734	5069	20692	7729	338	467	694	747	3624	2572	2675	1456	1357	1769	1208	1771	1062
DCAF4L2	3762	6835	1593	4324	2285	1113	4324	2335	26844	6049	15307	7246	604	504	831	581	5912	3823	4760	1125	1183	1367	904	1549	968
DCLK1	1651	2113	389	1146	575	404	1183	642	7336	1523	6907	1854	184	212	222	189	1185	718	914	463	435	558	446	740	383
DCSTAMP	6973	5164	1201	3445	2126	1091	3218	1832	20808	5035	11922	5972	427	513	550	576	3764	2672	2930	1144	1137	1357	861	1612	956
DDI1	3450	5625	1706	4285	2758	1444	3902	2166	28348	6743	15640	6023	580	609	848	744	12828	10680	11134	3733	3761	3656	5305	7114	4746
DLGAP2	0	0	0	0	0	0	0	0	0	1	0	0	0	0	0	0	1	0	0	0	0	0	0	0	0
DMD	682	721	206	890	516	202	1023	405	4511	843	3679	1635	107	90	117	130	1150	853	855	309	260	303	136	435	170
DNTTIP1	822	882	201	511	195	185	885	636	3580	1451	3071	1275	53	80	103	65	1639	1424	1248	957	1179	710	455	629	415
DOCK3	1470	1924	384	719	307	254	975	675	5143	1374	2748	1392	117	99	201	148	1773	1246	1475	1594	1514	1544	2049	2480	1806
DPYD	2925	3115	1013	2615	1850	927	2491	1762	17699	3863	9499	4296	297	345	509	418	2993	2238	2232	3356	3728	3384	1727	3152	1602
DSC3	715	746	259	555	378	184	830	594	3047	1138	2987	1423	86	111	153	60	1339	1193	1093	623	593	581	138	260	95
DSCAM	1385	1323	373	826	346	194	972	648	3745	1504	4183	1498	153	108	195	167	750	473	399	216	214	268	173	254	129
EGFLAM	1214	1617	268	677	353	235	729	446	5729	1601	4055	1319	149	117	177	87	1904	1454	1485	366	376	472	333	460	254
EGFR	72336	39086	9175	31998	16767	8557	23513	14012	142994	34707	99143	41482	3665	3543	4775	3849	395003	323450	348997	7048082	7543550	7839849	2375097	2687753	2327233
EPHA5	1679	2573	545	1445	664	397	1800	820	8696	2770	5148	2945	214	225	221	255	3694	2822	2773	1090	1155	1019	715	1369	608
EPHA6	2136	2482	611	1770	733	426	1839	981	9147	2775	7646	3085	286	221	398	270	2975	2241	2047	1546	1700	1571	1123	2018	1021
ERBB2	56213	46965	11386	24360	16723	8406	26607	13754	158242	46881	107785	41947	4263	4480	5907	5174	277016	216543	238210	151828	154769	162781	172286	190002	165293
EYS	2364	3065	1001	2215	1363	594	2496	1412	12729	2817	11083	4111	284	287	426	272	3038	2353	2139	773	736	841	512	1181	477
FAM135B	7698	10459	2267	6107	3057	1709	5824	3387	40011	10688	19368	11283	853	917	1260	1027	9544	7002	6921	1619	1325	2043	1253	2131	1004
FAM151A	974	1382	360	804	327	221	713	502	4795	1392	3177	1187	150	229	183	123	767	545	585	505	496	660	504	634	324
FAM71B	3872	6942	1375	3488	1591	1100	3485	1926	21778	6668	11479	5676	589	785	677	560	5018	3225	3575	1492	1336	1545	1016	1678	1009
FAT1	1404	1889	500	1119	574	300	1373	913	6126	1585	4660	2391	128	127	315	241	12519	10115	11128	6689	6990	7097	4538	5681	4639
FBN2	1844	2485	641	1751	815	520	1761	1208	10223	2383	5159	2785	268	237	358	268	8767	7201	7571	1379	1527	1362	753	1468	688
FBXL7	8702	7415	1776	4008	2270	1477	4514	2445	31212	7673	23956	8426	606	614	1065	786	6576	4686	5201	1038	1023	1224	679	1327	819
FBXW7	2314	2601	780	2321	1792	1065	2093	1322	16375	2351	11314	3840	185	324	443	420	12536	11436	11278	7887	9123	7617	4504	5870	4645
FCRL5	1489	1333	380	759	311	334	794	390	4982	2104	3822	1386	109	131	147	137	1008	852	689	381	415	486	320	584	263
FOXG1	4727	5462	1651	4164	2277	1395	3240	2076	22183	3472	37268	5104	522	468	952	667	3292	2808	2651	1960	2150	2000	1875	2863	1617
FRYL	1014	1183	392	786	639	314	814	598	6239	1116	4929	1902	64	114	131	115	4940	4158	4303	9376	10178	9902	5632	7699	5503
GALNT17	1388	1553	382	910	493	262	834	518	5436	1208	3617	1566	78	149	166	143	1419	1114	1117	245	215	295	145	347	120
GBA3	1708	2566	666	1703	880	525	1599	850	9750	2561	6109	2747	284	243	312	227	1829	1210	1132	1143	1142	1303	850	1704	802
GBP7	815	1347	354	1062	517	310	908	649	5619	1405	4395	1312	76	102	120	131	861	593	552	1208	1120	1301	731	1260	831
GJA8	7472	6033	1370	3674	1786	1087	3519	2018	21654	6621	16424	5271	455	573	703	485	3634	2466	2830	1478	1432	1753	1236	2226	1170
GPR139	2567	4468	811	2394	1132	680	2412	1551	14756	3909	12596	4392	446	433	546	338	3403	2573	2853	895	894	1080	711	1217	557
GRIA2	1087	1164	362	862	438	249	1000	487	4827	1134	3102	1460	142	93	127	156	996	603	603	606	631	721	329	759	309
GRIK3	1361	1933	420	919	333	348	927	651	5179	1992	8439	2017	229	203	275	181	654	438	511	299	291	238	188	322	203
GRIN2B	1175	2507	462	1417	612	422	1339	736	8198	2152	5502	2434	258	224	236	246	8050	6639	6948	730	718	932	595	1000	555
GRIN3B	2026	3674	635	1662	813	514	1878	946	9288	4355	9642	3353	257	259	460	277	5347	4318	4615	898	906	932	921	1349	678
GRM1	3285	5355	1108	3049	1317	853	3131	1872	17967	4183	11330	4782	518	429	661	448	2595	1554	1966	908	873	1016	963	1924	840
GRM5	2336	4113	1084	2338	1138	759	2400	1449	15836	4300	9810	4149	334	344	452	396	3073	2082	2325	1610	1683	1852	2364	3872	2069

GRM8	3044	4585	1055	2790	1476	785	2850	1757	18016	3745	19389	5362	318	457	675	430	4070	3164	3069	1433	1343	1623	818	1718	784
GSX1	709	1636	380	822	440	239	780	551	3947	1053	3585	1027	83	113	274	103	495	364	367	203	172	223	229	248	160
HACD1	695	1373	284	577	221	213	573	465	4557	913	2378	828	64	119	164	102	864	779	636	168	197	152	87	106	112
HCN1	6388	6157	1532	3590	1577	945	4253	2784	19354	7183	35957	6408	578	504	821	585	5369	4093	4295	1286	1583	1384	2031	2755	1666
HCRTR2	1186	1001	262	742	363	203	606	459	3922	862	2750	1180	103	176	81	100	840	588	624	596	678	637	338	606	284
HEBP1	1119	1521	409	996	665	366	1172	389	7366	1308	6272	1718	97	165	243	137	10622	8774	9671	8152	8305	8848	6685	7444	6170
HECW1	7366	7872	1443	3653	1698	994	3659	2088	21188	6924	14520	5987	601	643	741	654	24934	19421	21340	1311	1415	1812	1138	1818	1161
HS3ST4	2095	3576	813	2012	1086	672	2128	1192	12329	3304	7477	3350	265	330	456	349	2778	1865	2002	448	452	628	303	762	366
HS3ST5	3282	4017	967	2729	1466	738	2698	1678	19665	3560	11741	4476	350	391	546	426	2666	1751	1769	3257	3397	3352	1466	2350	1419
HTR1A	4013	7316	1925	4941	2480	1501	4773	2552	25875	5561	13563	8162	521	550	946	553	4985	3522	3662	1092	914	1072	828	1203	711
HTR1E	3346	6008	976	2989	1571	927	3113	2012	21077	4532	12885	4852	511	488	780	458	2671	1842	2037	1018	936	1362	797	1487	713
HTR2C	2754	1126	232	1486	738	406	1374	683	8873	1194	6337	2512	111	132	163	185	1178	808	892	628	681	840	369	800	415
IFI16	2028	1071	440	890	783	391	984	765	7000	1304	3823	1900	82	75	200	116	1897	1485	1419	7273	8626	8022	5037	7016	5074
IL7R	2189	2426	537	1349	722	485	1303	756	9000	2508	6313	2201	178	201	314	197	1583	1022	1174	2986	3231	2991	1486	2338	1304
INSL3	951	1236	339	852	285	215	693	369	4535	1751	2949	1159	90	122	142	113	611	435	472	349	355	407	312	455	250
ITGA10	2491	1764	515	1158	759	323	1168	432	7126	1705	4615	1597	122	125	251	201	1867	1466	1379	628	641	821	684	1090	602
ITSN1	1855	1393	404	1108	579	386	1197	594	7227	1738	7996	2190	144	164	261	170	4920	4615	4750	3266	3528	3305	1657	2309	1668
KCNA5	3050	7707	1699	3574	1601	1115	4049	1899	21975	6868	15288	6791	806	682	932	831	2965	1783	2028	1712	1551	2001	1426	2249	1229
KCNB2	2161	3002	642	1830	935	550	1947	1249	12989	2924	15401	3812	354	283	357	286	2111	1410	1552	1371	1321	1802	1014	2064	960
KCNC2	2112	2783	705	1763	961	530	1765	925	11090	3463	5604	2975	269	259	254	331	5385	4391	4278	1935	2065	2022	1379	2517	1286
KCNJ3	1787	1907	592	1406	742	470	1439	945	9877	2318	4871	2299	175	225	265	229	2142	1638	1502	863	825	986	671	1262	643
KCTD8	1442	2044	426	1366	590	361	1510	901	7063	2183	3839	2441	148	240	232	215	1873	1431	1389	839	762	1067	515	1151	545
KEAP1	10426	8357	2865	6667	5426	2593	7014	3179	54576	10333	75067	12261	675	793	1280	965	77355	62340	64843	53017	55968	57476	45446	50767	44767
KIF17	566	1352	262	714	263	233	635	514	3653	1197	2589	1209	74	63	127	60	1309	1193	1153	302	270	233	121	277	80
KIF19	1014	1501	279	568	173	233	596	272	4673	2475	6555	1498	94	88	167	131	728	525	549	133	151	149	115	198	142
KIT	19289	19546	5524	14606	8109	4518	13706	7607	90500	20924	88837	28854	2107	2019	3055	2075	12779	9083	8279	3589	3623	4762	2499	5339	2586
KLHL31	1060	2285	329	662	284	206	1172	669	5359	1365	2527	1103	86	91	126	92	527	341	388	106	150	112	38	150	70
KPRP	9967	11828	2019	4851	2226	1569	5633	3449	32901	13068	20659	7807	852	998	1498	961	4622	2880	3276	1623	1447	1951	1345	2557	1366
KRAS	3618	4280	1479	3581	2485	1350	3933	2431	24669	5493	19003	7422	597	542	681	555	33205	30614	26205	28553	34819	29746	14062	19416	14331
LRFN5	5567	5482	1487	3762	1632	1024	3560	2243	21170	4530	26853	7858	515	508	657	475	6551	5042	4856	2932	2666	3561	2451	4047	2117
LRP1B	975	1436	453	1184	395	340	955	737	4652	1288	3157	1714	111	99	168	95	1451	1090	1043	645	638	631	380	826	387
LRRC7	4939	7593	1929	5341	3000	1547	4814	2853	37501	8286	37981	9209	630	756	1201	719	4446	3181	3492	6344	6382	6462	3850	6255	3339
LRRTM1	4748	8581	1927	4360	1898	1280	4228	2434	23542	7039	17436	7063	574	595	1045	634	6223	4789	4996	1457	1457	1742	1188	1878	1046
LRRTM4	4538	6941	1706	4598	2397	1190	4655	2667	25349	6492	17940	8211	508	722	921	632	10483	7907	7179	2146	2232	2763	1871	3854	1559
LTBP4	949	1356	372	683	288	202	661	406	4161	1018	3543	1163	128	71	216	178	5232	4423	4423	223	184	231	144	243	154
MAP2	1700	1709	382	878	368	359	1104	686	6056	1757	8995	2271	98	252	231	141	1839	1191	1273	688	756	878	710	1228	649
MAP7D3	2753	1141	288	1531	1038	636	1575	729	8565	1141	5636	2188	77	94	184	208	11608	9389	9710	8842	9143	10218	7202	8132	7000
MET	31496	30113	7629	20986	12166	6957	21010	12602	127328	26900	115687	35767	2771	3243	4088	3096	380309	343191	335303	323145	379599	332205	938166	1217435	1001535
MKRN3	3811	8700	2030	4291	2006	1195	4415	2638	26954	6297	22172	5612	678	724	1094	644	18896	15683	16212	1275	1215	1522	994	1587	954
MMP16	921	1124	341	844	366	209	845	463	5125	1164	3203	1514	145	123	199	166	1679	1109	1078	310	331	437	162	384	184
MTX1	1556	947	246	490	194	146	664	484	3616	1828	2786	922	61	88	101	84	1234	1064	1012	698	841	648	364	522	277
MYH7	962	1498	399	835	445	270	966	494	4843	1125	3947	1437	81	180	164	214	1018	786	771	276	276	359	299	505	268
MYT1L	1062	1707	384	870	431	214	771	530	5405	2000	6680	1635	192	167	226	195	2014	1298	1288	478	448	594	336	577	267
NAV3	3503	5044	1235	3107	1839	829	3642	1955	22065	5965	12051	5036	450	496	572	449	14018	11004	11855	19021	19939	21316	14136	16764	13983
NEUROD4	3181	4983	1239	2845	1438	933	3020	1576	23417	3965	16387	4329	372	520	520	522	3453	2453	2397	1128	1137	1603	902	1681	1036
NFE2L2	966	953	324	783	693	252	802	376	5877	998	4770	1528	95	99	197	144	14747	12997	13361	10739	12076	11246	6647	8314	6764
NLGN4X	1325	2267	375	1756	833	522	1526	644	11141	1698	9168	3580	72	172	215	265	2015	1258	1427	506	513	566	287	584	329
NLRP3	7674	9112	2663	6695	4755	2466	6508	3239	50736	8584	28951	9093	937	801	1243	1079	7314	5505	5493	15110	15255	16806	8933	10590	8397
NMUR1	1319	2030	476	1179	547	357	1090	551	7424	2015	4240	1972	154	175	194	178	1370	898	1146	473	428	518	407	610	403
NOL4	719	1774	398	926	512	289	1149	667	5200	1523	7580	1487	92	158	262	144	717	537	638	312	322	239	78	131	47
NPAP1	8303	18649	3714	10199	4855	3059	9697	5872	62778	16942	42423	14297	1381	1666	2153	1548	17013	12116	13014	1896	1775	2305	1563	2828	1613
NR0B1	1775	2885	473	2397	1134	568	2135	790	14393	2334	9226	3065	232	215	301	398	5297	5051	4679	995	1010	985	780	1051	671
NRAS	4147	4675	1298	3594	2685	1356	3775	2213	24255	4287	24001	6519	449	478	712	438	26722	23946	24552	26121	28417	27320	16493	19565	16503
NRXN1	1573	2346	684	1874	1031	493	1637	801	11606	2331	20983	4404	253	183	414	365	3253	2277	2581	619	597	757	504	805	436
NXPH4	1481	2060	454	1007	622	339	1388	647	6601	1842	11680	2158	146	204	204	120	2006	1709	1718	834	896	893	3459	4008	3279
NYAP2	4638	5218	1283	3305	1308	876	3528	2015	19603	5528	20010	5925	375	503	733	477	3517	2394	2672	8316	8367	8618	2322	3317	2090
OPRD1	499	1558	316	565	275	170	404	282	3464	1229	1752	745	111	118	131	137	324	249	207	132	119	141	131	223	113
P2RY10	5002	3293	861	3892	2063	1121	3590	1749	25726	2895	12980	5091	268	354	498	593	3257	2282	2255	1502	1530	1969	768	1865	937
PAX6	778	1570	358	949	456	309	854	471	6088	1709	10770	1827	101	113	214	149	2796	2086	2309	1778	1766	1902	1737	2269	1792
PCDH15	4783	6271	1709	4147	1933	1150	5393	3463	22826	6535	16638	7780	657	727	950	695	7194	5234	4887	1139	1146	1574	882	1657	854
PDGFRA	21162	28458	7055	18597	10471	5567	17607	10445	107329	27244	60847	30472	2482	2539	3694	2866	17444	12364	12142	5906	5642	7091	4042	7654	3982
PDYN	2097	3513	694	1661	706	548	1642	1211	11271	3417	7637	2712	235	363	447	289	2175	1286	1589	552	532	700	492	752	454
PDZRN3	3482	6491	1436	3622	1956	1165	3467	1719	25462	4921	33658	5948	611	564	839	569	3778	2672	3010	795	775	1066	757	1201	603
PGK2	4712	5955	1534	4394	2843	1387	3844	1725	29437	4970	14141	5342	464	536	677	570	3208	2273	2224	1034	859	1297	949	1737	986
PHACTR1	2060	1691	584	1215	484	406	830	457	6951	2247	8281	2625	92	163	212	200	854	603	621	276	189	244	218	384	217
PIK3CA	3477	3314	924	2769	1917	1180	2797	1696	18163	4812	23140	6157	270	356	481	417	9853	8657	8084	11152	12424	11363	6343	9287	6608
PIK3CG	7534	8362	2955	7113	5881	2682	7722	3534	56584	7238	29317	10974	828	868	1239	1023	5999	4064	4669	2762	2643	3106	3326	5376	2988
PKHD1L1	5044	3842	1016	2622	1602	879	2967	1638	16834	4131	9333	5896	373	355	525	335	3653	2454	2379	1110	995	1263	982	1804	955
PLPPR4	4431	6640	1496	3621	1791	964	3597	1933	25599	6559	23485	7427	617	639	667	525	2923	2168	1827	1882	1816	2236	1612	2731	1320
POLE	2283	2072	771	1343	1280	423	1778	674	12007	2763	12051	2415	216	210	326	295	14168	10548	12552	6365	6276	6979	4352	4669	4191
POM121L12	3701	4576	1148	2034	905	766	2098	1494	14478	4346	8592	3476	309	404	459	360	3653	2756	2868	757	687	875	594	924	496
PREX1	1580	1760	610	1500	945	492	1747	885	10706	1749	6481	2361	155	205	236	288	6447	5239	5898	314	294	405	368	485	299
RALYL	668	838	219	661	245	189	727	391	3641	971	3889	1815	103	87	116	82	1514	1304	1034	282	248	347	124	265	136
RET	24131	44063	9770	21841	13739	7317	23770	14652	142481	37391	121492	35141	3576	3999	4952	3919	19706	12557	14295	4580	4259	5382	4022	6700	3690
RFX5	3038	1689	367	767	561	262	1081	616	5800	1826	6999	1656	100	112	143	142	8776	7615	8093	3991	3994	3691	1510	2491	1560
RIN3	1889	1294	571	1313	969	534	1363	675	10268	967	3981	1548	114	152	239	194	5486	4234	4456	2365	2613	2400	2299	2456	2014
RNASE3	785	1064	309	680	314	207	757	363	3661	892	3039	1412	188	169	155	103	1082	687	649	450	414	595	355	527	320
ROBO2	726	944	317	614	289	162	663	305	3542	767	2160	916	104	118	174	224	1457	1068	1067	313	306	358	286	529	247
ROS1	56363	70409	16295	45711	21277	13609	55633	36090	239827	62117	166629	79224	6456	7094	9606	7055	44914	29910	27218	15685	15272	18773	11160	21678	11306
SEMA5B	1322	1396	351	704	442	172	700	416	4209	1504	5592	1562	111	129	188	159	493	295	385	287	312	290	294	503	249
SLC18A3	5202	7829	1774	4445	2216	1474	3315	1945	25382	5855	21315	6108	627	565	995	721	3311	2349	2699	1025	990	1147	1044	1451	849
SLC39A12	839	1230	418	1010	448	271	1189	788	4721	1384	3456	2030	264	240	262	183	1644	1248	1050	246	309	213	90	241	106
SLC6A5	1214	2178	568	1004	461	375	1125	524	6457	2162	5730	1919	190	235	299	240	1305	811	949	310	320	342	329	399	260

SLC8A1	5756	8147	2040	6017	3413	1866	6531	3350	42666	7746	35359	10236	734	825	1140	767	14039	10661	11238	12642	13094	13175	6192	9423	5566
SLITRK1	10351	17647	4506	11048	5729	3711	11799	7177	56274	15610	27518	17545	1575	1830	2183	1766	7733	5289	5590	3410	3308	3414	2666	5037	2662
SLITRK4	11680	6349	1498	7211	3443	2086	6908	3633	45642	6527	40520	11093	609	611	752	1034	5982	3835	3792	3694	3353	4394	2114	4150	1933
SLITRK5	7452	13368	3151	8234	4065	2697	7636	4481	47122	12704	34491	13107	1211	1207	1775	1279	24019	20213	21524	4802	4888	4680	3226	4790	2625
SLPI	1116	1045	286	799	554	197	777	397	4643	1308	3228	1328	98	84	127	107	2408	2222	2347	494	509	537	557	900	521
SMAD4	800	1046	336	828	637	371	807	464	7462	1132	9986	2183	69	149	157	110	8807	7639	7200	3448	3991	3734	1778	2218	1982
SOX9	4829	3775	974	2022	1325	658	2353	1333	12526	4769	26352	3863	341	368	540	417	33849	26888	29334	13593	16204	15614	15914	15967	14484
SPTA1	1671	1435	341	1057	683	429	1013	515	7301	1677	3663	1443	175	175	255	159	1219	874	863	490	458	565	264	561	275
ST6GALNAC3	797	1072	303	844	478	293	822	519	5717	1066	3909	1628	102	112	182	126	991	541	601	598	552	594	386	705	321
STK11	3450	2501	864	1893	1136	685	2278	1196	12864	3637	16901	3481	211	207	422	230	15137	13512	13362	5987	6223	5702	3975	4327	4026
SV2A	2380	1608	557	1174	883	413	1480	995	7888	2430	13064	2795	85	127	208	235	1592	985	1293	558	524	647	620	777	510
TBXT	1455	2087	480	997	370	308	903	623	5819	1771	4478	2014	146	226	181	194	1206	820	932	321	277	445	263	473	273
THSD7A	4744	3637	844	2337	1076	744	2148	1225	12903	3056	8929	3685	301	271	485	284	15743	13541	14509	2733	2835	2649	1516	2431	1426
TIAM1	6218	5030	1313	3243	2086	1104	3098	1639	19481	4866	25299	5197	451	449	612	507	3223	2356	2497	7948	8090	8324	8499	10073	8538
TMEM200A	3720	6373	1627	4704	2423	1442	4009	2183	27340	5225	14857	6246	557	560	902	544	3348	2343	2390	1443	1376	1723	879	1815	919
TNFRSF21	3162	2390	701	1674	951	637	1489	989	11310	2142	10195	2581	186	276	225	287	18753	15807	16502	14861	15255	15429	10056	11554	10094
TNN	2030	2027	449	890	351	262	1133	395	5957	2511	4254	1518	264	203	222	229	1142	758	762	539	532	550	451	715	427
TNR	4314	5237	1149	2499	1198	668	2880	1377	17579	5866	12405	4094	284	474	676	397	2819	1695	1679	978	926	1176	918	1448	794
TP53	15895	15019	6221	15508	13282	6760	13833	6867	110529	17261	98735	17923	1590	1575	2792	2170	225416	189132	196345	108552	111108	119880	122970	137383	116959
TRHDE	983	1258	317	955	484	287	880	472	5271	1454	3920	1563	111	83	184	115	1787	1214	1310	1489	1663	1344	1490	2180	1430
TRIM58	2467	3412	1006	2441	1778	897	2643	1364	18873	2996	8985	3136	311	309	546	419	2911	2183	2223	904	752	1047	516	1172	616
TRPS1	9156	5619	1742	4313	3579	1958	5102	2847	33881	5549	14062	8087	470	690	923	579	4673	2938	3401	15272	15286	15503	12338	15495	12248
UGT1A1	625	996	215	483	230	178	548	292	2842	1049	1992	916	76	110	151	127	4692	4494	4584	388	311	471	269	486	237
UGT3A2	1200	1587	393	857	315	227	991	554	5508	1667	4616	1358	146	175	185	141	1082	704	695	274	256	341	222	371	305
USH2A	3382	4122	1036	2685	1256	914	2606	1640	16671	4258	11849	4115	401	435	558	389	3721	2681	2639	1369	1331	1751	1008	2072	1033
USP29	6895	10996	2327	6887	3852	2069	6274	3475	45500	8356	28153	10431	856	1119	1422	1075	15948	11982	11597	4282	4288	5022	2703	5304	2753
VPS13B	1763	1013	352	752	597	301	941	657	5354	1015	2994	1438	69	87	140	136	2571	2177	2172	920	1017	989	726	1153	766
WIPF1	3991	2748	1122	2852	2207	989	2782	1257	19183	3058	14711	4188	286	245	400	507	9115	7553	8295	931	813	967	795	1146	673
WSCD2	698	1224	290	395	145	120	589	311	3043	1301	2353	960	68	149	107	95	535	346	399	184	198	245	191	334	222
ZC3H12A	1767	1554	498	1323	1039	537	1317	645	9039	1451	10633	1712	116	121	272	195	14349	12437	11969	8811	9459	9285	7026	7990	7108
ZFPM2	19892	11007	3630	9958	7489	4075	7913	4885	62187	10095	27317	14298	1050	1162	1559	1199	35528	28397	31286	17307	17839	18460	16559	20695	15889
ZIC1	4081	6047	1598	4274	2314	1291	3814	2116	23676	6744	22048	7763	520	522	961	619	3842	2635	2963	1547	1610	1748	2092	3205	1693
ZIC4	2966	4168	1134	2790	1636	917	2690	1456	16651	5489	14538	5625	451	431	639	459	2558	2090	2303	977	874	912	1461	2040	1196
ZNF521	8146	16988	4085	10601	6213	3395	10155	5503	69210	16938	52768	23828	1373	1552	2117	1461	7471	4519	5214	2551	2508	3307	988	1686	1103
ZSCAN1	1101	1796	529	1010	438	350	1107	516	7214	1489	5200	1656	85	228	329	194	2500	1912	2019	579	529	563	424	637	427
